# The association between perinatal factors and cardiometabolic risk factors in children and adolescents with overweight or obesity: A retrospective two-cohort study

**DOI:** 10.1371/journal.pmed.1004165

**Published:** 2023-01-13

**Authors:** Nicole Prinz, Resthie R. Putri, Thomas Reinehr, Pernilla Danielsson, Daniel Weghuber, Mikael Norman, Niels Rochow, Claude Marcus, Reinhard W. Holl, Emilia Hagman

**Affiliations:** 1 Insitute of Epidemiology and Medical Biometry, ZIBMT, University of Ulm, Ulm, Germany; 2 German Center for Diabetes Research (DZD), Munich-Neuherberg, Germany; 3 Division of Pediatrics, Department of Clinical Science, Intervention and Technology, Karolinska Institutet, Stockholm, Sweden; 4 Department of Pediatric Endocrinology, Diabetes and Nutrition Medicine, Vestische Hospital for Children and Adolescents Datteln, University of Witten/Herdecke, Datteln, Germany; 5 Department of Pediatrics, Paracelsus Private Medical School, Salzburg, Austria; 6 Obesity Research Unit, Paracelsus Private Medical School, Salzburg, Austria; 7 Department of Pediatrics, Paracelsus Medical University, Nuremberg, Germany; 8 Department of Pediatrics, University Medical Center Rostock, Rostock, Germany

## Abstract

**Background:**

Children with obesity have an increased risk of cardiometabolic risk factors, but not all children carry a similar risk. Perinatal factors, i.e., gestational age (GA) and birth weight for GA, may affect the risk for metabolic complications. However, there are conflicting data whether the association between birth size and cardiometabolic risk factors is independent among children with obesity. Moreover, differential effects of GA and birth weight for GA on cardiometabolic risk factors in pediatric obesity are still unexplored. We aimed to investigate the association between birth weight for GA and cardiometabolic risk factors in children and adolescents with overweight or obesity and to assess whether the association is modified by prematurity.

**Methods and findings:**

We conducted a retrospective study of 2 cohorts, using data from the world’s 2 largest registers of pediatric obesity treatment—The Swedish childhood obesity treatment register (BORIS) and The Adiposity Patients Registry (APV) (1991 to 2020). Included were individuals with overweight or obesity between 2 to 18 years of age who had data of birth characteristics and cardiometabolic parameters. Birth data was collected as exposure variable and the first reported cardiometabolic parameters during pediatric obesity treatment as the main outcome. The median (Q1, Q3) age at the outcome measurement was 11.8 (9.4, 14.0) years. The main outcomes were hypertensive blood pressure (BP), impaired fasting glucose, elevated glycated hemoglobin (HbA1c), elevated total cholesterol, elevated low-density lipoprotein (LDL) cholesterol, elevated triglycerides, decreased high-density lipoprotein (HDL) cholesterol, and elevated transaminases. With logistic regression, we calculated the odds ratio (OR) and its 95% confidence interval (CI) for each cardiometabolic parameter. All the analyses were adjusted for sex, age, degree of obesity, migratory background, and register source.

In total, 42,760 (51.9% females) individuals were included. Small for GA (SGA) was prevalent in 10.4%, appropriate for GA (AGA) in 72.4%, and large for GA (LGA) in 17.2%. Most individuals (92.5%) were born full-term, 7.5% were born preterm. Median (Q1, Q3) body mass index standard deviation score at follow-up was 2.74 (2.40, 3.11) units. Compared with AGA, children born SGA were more likely to have hypertensive BP (OR = 1.20 [95% CI 1.12 to 1.29], *p* < 0.001), elevated HbA1c (1.33 [1.06 to 1.66], *p* = 0.03), and elevated transaminases (1.21 [1.10 to 1.33], *p* < 0.001) as well as low HDL (1.19 [1.09 to 1.31], *p* < 0.001). On the contrary, individuals born LGA had lower odds for hypertensive BP (0.88 [0.83 to 0.94], *p* < 0.001), elevated HbA1c (0.81 [0.67 to 0.97], *p* < 0.001), and elevated transaminases (0.88 [0.81 to 0.94], *p* < 0.001). Preterm birth altered some of the associations between SGA and outcomes, e.g., by increasing the odds for hypertensive BP and by diminishing the odds for elevated transaminases. Potential selection bias due to occasionally missing data could not be excluded.

**Conclusions:**

Among children and adolescents with overweight/obesity, individuals born SGA are more likely to possess cardiometabolic risk factors compared to their counterparts born AGA. Targeted screening and treatment of obesity-related comorbidities should therefore be considered in this high-risk group of individuals.

## Abstract 2

In a retrospective study of two cohorts, Dr. Nicole Prinz, Dr. Resthie R Putri and colleagues investigate the association between perinatal factors and cardiometabolic risk factors in children and adolescents with overweight or obesity.

## Author summary

**Why was this study done?:**

- Pediatric obesity and born small for gestational age (SGA) are 2 risk factors for cardiometabolic disorders.
- There are conflicting data regarding whether the association between birth size and cardiometabolic risk factors in childhood is independent or mediated by adiposity.
- Differential effects of SGA and preterm birth on cardiometabolic risk factors in pediatric obesity are unknown.

**What did the researchers do and find?:**

- Using data from the world’s 2 largest registers of pediatric obesity treatment, we investigated the association between SGA and cardiometabolic risk factors among children with overweight or obesity and explored whether the association between SGA and cardiometabolic risk factors was modified by preterm birth.
- Compared with appropriate for gestational age (GA), children with overweight/obesity born SGA were, independent of sex, age, and degree of obesity, more likely to have elevated blood pressure, glycated hemoglobin (HbA1c), and transaminases as well as low high-density lipoprotein (HDL) cholesterol.
- Preterm birth modified some of the associations between SGA and outcomes, e.g., by increasing the odds for elevated blood pressure and by diminishing the odds for elevated transaminases.

**What do these findings mean?:**

- In pediatric obesity treatment, early screening and treatment of obesity-related comorbidities should be considered in children who are born SGA.

## Introduction

Childhood obesity remains an important challenge in global health. The number of children aged 5 to 19 years with obesity is predicted to rise to 254 million by 2030 [1]. Children with obesity have an increased risk of cardiometabolic risk factors, such as hypertension [2,3], dyslipidaemia [3], impaired glucose metabolism [4], and non-alcoholic fatty liver disease (NAFLD) [5,6]. However, not all children with obesity develop associated comorbidities, suggesting that other factors than obesity may contribute to adverse outcome. Previous research has shown differences between age, sexes, and ethnicity [2–4]. Perinatal factors, i.e., gestational age (GA) and weight for GA may also contribute. Several studies have indicated a positive association between being born small for GA (SGA) and an increased risk for elevated blood pressure (BP) [7–9], type 2 diabetes [10,11], dyslipidaemia [10], and NAFLD [12] in childhood and adolescence. However, due to different study designs, there are conflicting data whether the association between birth size and cardiometabolic risk factors is independent or mediated by adiposity [7,10,12,13]. Moreover, some studies used or interpreted GA and birth weight for GA interchangeably [10,11]. However, SGA and preterm birth might be associated with cardiometabolic factors in different ways and underlying mechanisms [14].

Childhood obesity is per se a risk factor for cardiometabolic diseases [15] and to which extent perinatal factors contribute to the risk for cardiometabolic comorbidities in children with obesity remains unclear. Evidence in children with overweight/obesity is scarce and inconsistent [16–19]. For example, one study found an association between SGA and hypertension [16], while the other study reported no association [17,18]. These previous studies were limited by rather small numbers of individuals born SGA and the possibility of selection bias, as the children were recruited from single specialist centers. A paradox is that children born SGA seem to be underrepresented in the pediatric obesity population, while children born large for GA increase the risk for obesity in childhood [19,20].

In the present study, we used longitudinal data from the 2 largest cohorts of childhood obesity in Europe to investigate the association of SGA or large for gestational age (LGA) with cardiometabolic risk factors in children and adolescents with overweight/obesity and to assess whether this association was modified by preterm birth. We hypothesized that SGA is associated with increased cardiometabolic risk factors in children and adolescents with overweight/obesity. We also hypothesized that such association could be altered by GA.

## Methods

**Fig 1:**
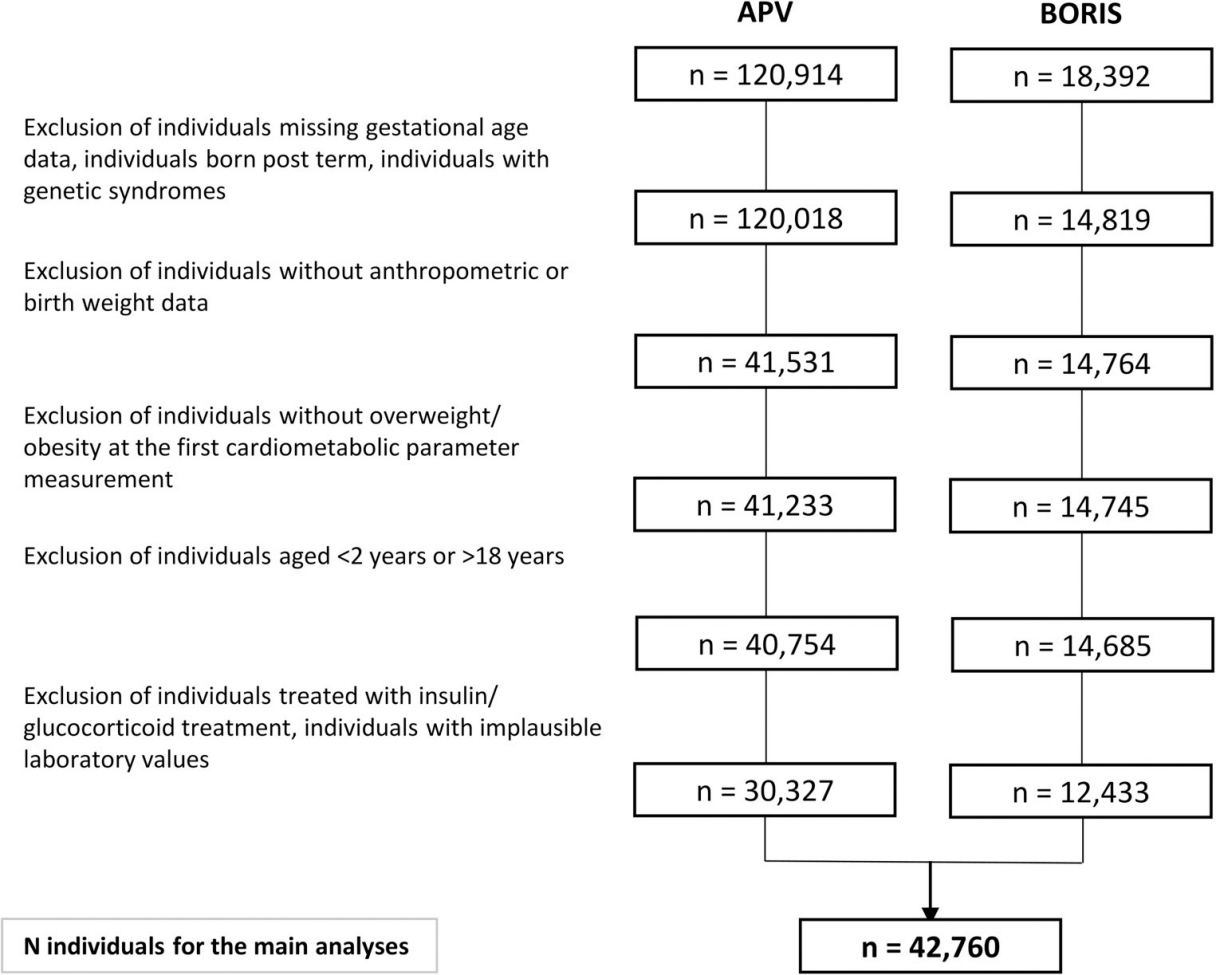
Flowchart detailing participant inclusion and exclusion for each childhood obesity register (APV and BORIS), respectively. APV, the German/Austrian/Swiss adiposity patients register; BORIS, the Swedish childhood obesity treatment register.

We conducted a retrospective two-cohort study, using data from the Swedish childhood obesity treatment register (BORIS) [21] and the German/Austrian/Swiss Adiposity Patients Registry (APV) [3]. A detailed description of the obesity registers can be found in the S2 File. Included were individuals with overweight or obesity seeking pediatric obesity treatment between 2 to 18 years of age with available data on birth characteristics and cardiometabolic parameters, such as BP, fasting glucose, glycated hemoglobin (HbA1c), lipid profile, and transaminases. To reduce the risk that other factors affect the exposure or outcome, some exclusion criteria were applied: insulin-treated diabetes, genetic syndromes, systemic glucocorticoid treatment, multiples (twins, triplets), and postterm birth (≥42 weeks). Flowchart of this process is presented in Fig 1.

The individuals were born between November 1977 and August 2018. Documentation of the first reported cardiometabolic measurement during pediatric obesity treatment was recorded between June 1991 and August 2020. Since we only included individuals who had cardiometabolic parameters data at follow-up, there was no loss of follow-up in the study. However, not all individuals had complete data of all cardiometabolic parameters.

This study is reported as per the Strengthening the Reporting of Observational Studies in Epidemiology (STROBE) guideline (S1 File). The analysis plan was developed a priori (S3 File).

### Ethical approval

For the Swedish data, ethical approval was obtained by the regional Ethics Committee in Stockholm, Sweden for linking individuals in BORIS with the Swedish medical birth register (No. 2016/922-31/1) and sending data to Ulm University, Germany (No. 2020–05646).

For the APV data, the Ethics Committee of the University of Ulm, Germany (No. 133/22) has approved the APV initiative and the local review boards have authorized documentation in the participating centers and the transfer of anonymized data to Ulm for benchmarking and research.

### Variables and definitions

#### Gestational age and birth weight for gestational age

GA was determined according to prenatal ultrasound or last menstrual period and categorized into preterm birth (before 37 completed weeks of gestation) and full-term birth (from 37 to <42 completed weeks).

Birth weight for GA was categorized as (i) appropriate for GA (AGA) defined as a birth weight for GA between the 10 to 90th percentile; (ii) SGA defined as <10th percentile; and (iii) LGA defined as >90th percentile using 2 national references for fetal growth from Sweden and Germany, respectively [22,23]. In the main text, data are presented based on the German (Voigt) reference and the corresponding data with the Swedish (Maršál) reference can be found in the S2 File.

For the Swedish cohort, data was retrieved from the Swedish Medical Birth Register (see S2 File for more information), and for the APV Cohort, data was retrieved from maternity records by the participating APV centers and entered into the standardized APV software. Hence, birth characteristics were prospectively collected.

#### Degree of obesity

Body mass index standard deviation scores (BMI SDSs) for age and sex were used to measure the degree of obesity in children and adolescents [24]. We classified the individuals into overweight, class I obesity, class II obesity, and class III obesity based on age- and sex-specific curves corresponding to adult BMI of 25, 30, 35, and 40 kg/m^2^, respectively [24,25]. Data was retrieved from APV and BORIS.

#### Age categories

Age at follow-up was categorized into 3 age groups and based on a subsample (*n* = 19,332) with data on pubertal status measured by Tanner stages. The prepubertal (Tanner = 1) age group were individuals <11 years of age, the pubertal (Tanner 2 to 4) group were 11 to 14 years, and the post-pubertal were ≥14 years. Data was retrieved from APV and BORIS.

#### Migration background

Individuals in BORIS were categorized as Nordic (born in, and with 1 or 2 parents born in, Sweden, Norway, Denmark, Finland, or Iceland) or non-Nordic. For individuals in APV, migration background was defined as children or either 1 parent being born outside Germany, Austria, or Switzerland. For individuals in BORIS, data was retrieved from Statistics Sweden, and for individuals in APV, data was retrieved from APV register.

#### Blood pressure and biomarkers

Data of blood pressure and biomarkers are entered into APV and BORIS by clinical staff from more than 200 pediatric obesity treating centers. Hence, the exact device for blood pressure measurement and analysis method for biomarkers may vary. However, the methodology and analysis of blood samples is regulated by “RILIBÄK” in Germany and by “EQUALIS” in Sweden. These policy frameworks advocate similar recommendations, e.g., venous plasma glucose when investigating fasting glucose level.

To compare BP in children, a sex, age, and height-adjusted reference from the US National High Blood Pressure Education Program (NHBPEP) Working Group was applied [26]. Hypertensive systolic or diastolic BP was defined as the 95th percentile or above for sex, age, and height. Since only 1 recorded blood pressure measurement was used, the term hypertensive BP is used instead of hypertension which would require additional measurements.

Lipid profile was categorized as follows: high total cholesterol ≥5.2 mmol/L (≥200 mg/dl); high low-density lipoprotein cholesterol (LDL) ≥3.4 mmol/L (≥130 mg/dl); low high-density lipoprotein cholesterol (HDL) <1.0 mmol/L (<40 mg/dl); high triglycerides ≥1.2 mmol/L (≥100 mg/dl) in individuals aged 5 to 9 years, or ≥1.6 mmol/L (≥130 mg/dl) in individuals aged ≥10 years [27].

Elevated alanine aminotransferases (ALTs) as a surrogate for infiltration of fat in the liver was set to ≥24 U/L (0.4 μkat/L) [28].

Dysglycemia was measured by fasting glucose and HbA1c. Impaired fasting glycemia (IFG) was defined as a fasting glucose ≥6.1 (110 mg/dl), and elevated HbA1c was defined as ≥39 mmol/mol (5.7%) [29,30].

### Statistical analyses

All statistics were carried out using SAS version 9.4 (Statistical Analysis Software, SAS Institute, Cary, North Carolina, United States of America). Descriptive analyses were performed for the whole study population as well as for birth weight for GA and both registries separately. Demographic and clinical data are presented as median (quartile 1 [Q1], quartile 3 [Q3]) or as proportion. Characteristics comparison between groups (SGA/AGA/LGA) were performed using Kruskal–Wallis test for continuous parameters and χ^2^-test for dichotomous parameters. In case of multiple comparisons, *p*-values were adjusted using Bonferroni–Holm method. Missing data were handled using pairwise deletion.

Logistic regression analyses, adjusted for age, sex, migratory background, degree of obesity, and register source were applied to study the association between birth weight for GA category and the following cardiometabolic outcome parameters: hypertensive BP, IFG, elevated HbA1c, elevated cholesterol, elevated LDL, elevated triglycerides, decreased HDL, and elevated ALT. GA was not included as a covariate in the main analyses given the collinearity between GA and birth weight for GA. Nevertheless, to explore the effect of GA on the association between birth weight for GA and cardiometabolic risk factors, interaction between GA and birth weight for GA was further included in each adjusted model. As significant interaction GA and birth weight for SGA was observed in some outcomes, the main analyses were stratified by GA category. In addition, stratified analyses by sex, age groups, degree of obesity, and register source were also performed. Odds ratios (OR), 95% confidence intervals (CIs), and *P*-values were calculated based on the logistic regression models. Two-sided *p*-values <0.05 were considered significant.

## Results

In total 42,760 (51.9% females) individuals were included of which 30,327 (53.5% females) originated from APV and 12,433 (48.0% females) originated from BORIS. Median (Q1, Q3) birth weight was 3,500 (3,140, 3,870) grams, and GA was 40 (38, 40) weeks. According to the Voigt reference, 10.4% were born SGA, 72.4% AGA, and 17.2% LGA. The corresponding frequencies for the Maršál reference were 12.0% SGA, 71.9% AGA, and 16.1% LGA. Most individuals (92.5%) were born full-term, and 7.5% (*n* = 3,201) were born preterm.

The individuals were followed from birth until the first recorded cardiometabolic measurements during pediatric obesity treatment as the end of follow-up. The median (Q1, Q3) age at follow-up was 11.8 (9.4, 14.0) years. The median BMI SDS was 2.74 (2.40, 3.11) units. The proportion of individuals with overweight was 15.1% (*n* = 6,442), obesity class I 44.6% (*n* = 19,071), obesity class II 25.5% (*n* = 10,908), and obesity class III 14.8% (*n* = 6,339). Migration background was present in 21.1% (*n* = 9,039) of the individuals. Characteristics by birth weight status are presented in Table 1 and more detailed characteristics by cohort are presented in S2 File. Unadjusted analyses for the association between birth weight for GA with each cardiometabolic outcome (S2 File) yielded similar estimates as the adjusted analyses. Stratification of the unadjusted associations by gestational age (full-term and preterm birth) is presented in S2 File.

**Table 1:** General characteristics stratified for birth weight status (Voigt).

	SGA *N* = 4,433 n/N (%)	AGA *N* = 30,952 n/N (%)	LGA *N* = 7,375 n/N (%)	p SGA vs. AGA	p LGA vs. AGA	All individuals *N* = 42,760 n/N (%)
Full-term birth	4,095/4,433 (92.4)	28,861/30,952 (93.2)	6,603/7,375 (89.5)	0.55	<0.001	39,559/42,760 (92.5)
Preterm birth	338/4,433 (7.6)	2,091/30,952 (6.8)	772/7,375 (10.5)	0.55	<0.001	3,201/42,760 (7.5)
Male sex	2,157/4,433 (48.7)	14,934/30,952 (48.3)	3,480/7,375 (47.2)	1.00	1.00	20,571/42,760 (48.1)
Migration background	923/4,433 (20.8)	6,692/30,952 (21.6)	1,424/7,375 (19.3)	1.00	<0.001	9,039/42,760 (21.1)
**Age category**						
<11 years	1,555/4,433 (35.1)	12,431/30,952 (40.2)	3,210/7,375 (43.5)	<0.001	<0.001	17,196/42,760 (40.2)
11 to <14 years	1,596/4,433 (36.0)	10,763/30,952 (34.8)	2,405/7,375 (32.6)	1.00	0.01	14,764/42,760 (34.5)
≥14 years	1,282/4,433 (28.9)	7,758/30,952 (25.1)	1,760/7,375 (23.9)	<0.001	1.00	10,800/42,760 (25.3)
**Degree of obesity**						
Overweight	787/4,433 (17.8)	4,875/30,952 (15.8)	780/7,375 (10.6)	0.015	<0.001	6,442/42,760 (15.1)
Obesity class I	1,958/4,433 (44.2)	13,972/30,952 (45.1)	3,141/7,375 (42.6)	1.00	0.002	19,071/42,760 (44.6)
Obesity class II	1,105/4,433 (24.9)	7,713/30,952 (24.9)	2,090/7,375 (28.3)	1.00	<0.001	10,908/42,760 (25.5)
Obesity class III	583/4,433 (13.1)	4,392/30,952 (14.2)	1,364/7,375 (18.5)	0.94		6,339/42,760 (14.8)
**Cardiometabolic risk markers**						
Hypertensive BP	1,465/4,066 (36.0)	8,522/27,940 (30.5)	1,832/6,547 (28.0)	<0.001	0.001	11,819/38,553 (30.7)
IFG	46/2,589 (1.8)	395/18,178 (2.2)	101/4,433 (2.3)	1.00	1.00	542/25,200 (2.2)
Elevated HbA1c	106/721 (14.7)	706/6,674 (10.6)	154/1,852 (8.3)	0.02	0.08	966/9,247 (10.5)
Elevated ALT	1,182/2,108 (56.1)	7,758/15,372 (50.5)	1,817/3,817 (47.6)	<0.001	0.03	10,757/21,297 (50.5)
Elevated triglycerides	753/2,901 (26.0)	4,985/20,255 (24.6)	1,125/4,848 (23.2)	1.00	0.61	6,863/28,004 (24.5)
Elevated total cholesterol	412/2,923 (14.1)	2,779/20,416 (13.6)	646/4,879 (13.2)	1.00	1.00	3,837/28,218 (13.6)
Elevated LDL	412/2,721 (15.1)	2,743/19,011 (14.4)	674/4,612 (14.6)	1.00	1.00	3,829/26,344 (14.5)
Low HDL	741/2,685 (27.6)	4,580/18,808 (24.4)	1,120/4,518 (24.8)	0.006	1.00	6,441/26,011 (24.8)

Birth weight status according to Voigt (German reference for birth weight) [23].

Obesity classification according to IOTF [24,25].

Hypertensive BP [26]: Any BP ≥95 percentile for age, sex, and height.

IFG [29]: ≥6.1 mmol/l respectively 110 mg/dl.

Elevated HbA1c [30]: ≥39 mmol/mol respectively 5.7%.

Elevated ALT [28]: ≥24 U/l respectively 0.4 μkat/l.

Lipids [27]: high total cholesterol ≥200 mg/dl; high LDL ≥130 mg/dl; low HDL <40 mg/dl; high triglycerides ≥130 mg/dl (10–19 years) or ≥100 mg/dl (0–9 years).

*P*-values were calculated using Kruskal–Wallis test for continuous parameters and chi-square test for dichotomous parameters, corrected for multiple testing using Bonferroni–Holm method.

ALT, alanine aminotransferase; AGA, appropriate for gestational age; BP, blood pressure; HbA1c, glycated hemoglobin; HDL, high-density lipoprotein; IFG, impaired fasting glycemia; IOTF, International Obesity Task Force; LDL, low-density lipoprotein; LGA, large for gestational age; SGA, small for gestational age.

### Hypertensive blood pressure

**Fig 2:**
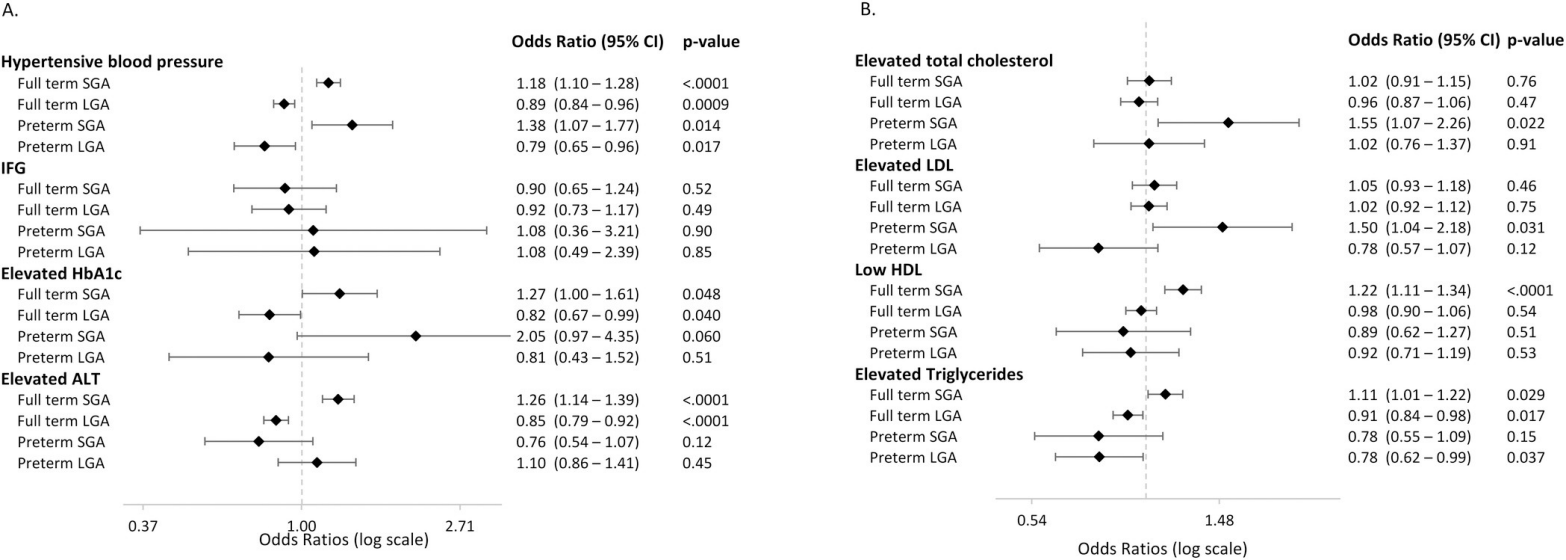
(A and B) ORs, 95% CIs, and *p*-values of SGA and LGA for cardiometabolic risk factors stratified by gestational age were calculated using logistic regression. Reference is AGA. Dashed line indicates OR = 1.00. Analyses were adjusted for sex, age category, degree of obesity, immigration status, and cohort origin. Unadjusted ORs are found in S2 File. ALT, alanine aminotransferase; AGA, appropriate for gestational age; BP, blood pressure; CI, confidence interval; HbA1c, glycated hemoglobin; HDL, high density lipoprotein; LDL, low density lipoprotein; LGA, large for gestational age; OR, odds ratio; SGA, small for gestational age.

Data on BP were available in 90.1% (*n* = 38,553) and the overall prevalence of hypertensive BP was 30.7% (*n* = 11,819). Compared to individuals born AGA, individuals born SGA were more prone to have hypertensive BP (OR = 1.20 [95% CI 1.12 to 1.29], *p* < 0.001), while individuals born LGA were less likely to have hypertensive BP (OR = 0.88 [0.83 to 0.94], *p* < 0.001), see Table 2. The risk for hypertensive BP increased with age, degree of obesity and was slightly higher in females. The greatest risk for hypertensive BP were belonging to the APV cohort with an OR of 2.58 [2.44 to 2.74], *p* < 0.001. The results were not attenuated when systolic and diastolic BP were investigated separately, see S2 File. Preterm birth was associated with systolic hypertensive BP (OR = 1.11 [1.02 to 1.21], *p* = 0.015), but not with diastolic hypertensive BP (OR = 1.06 [0.94 to 1.18], *p* = 0.338). No significant interaction between SGA and preterm birth on hypertensive BP (*p* = 0.256). Main analyses stratified for GA are provided in Fig 2.

### Glucose metabolism

Fasting glucose was available in 58.9% (*n* = 25,200) of all individuals and the overall prevalence of IFG was 2.2% (*n* = 542). In the adjusted model, neither SGA nor LGA altered the risk for IFG. Additionally, no interaction between SGA and preterm birth on IFG was found (*p* = 0.898). The risk for IFG increased with age and severity of obesity, but the single largest risk factor for IFG were belonging to the Swedish cohort, OR = 3.54 [2.96 to 4.25], *p* < 0.001.

Data of HbA1c was available in 21.6% (*n* = 9,247) of all individuals and the prevalence of elevated HbA1c was 10.5% (*n* = 966). Compared to individuals born AGA, elevated HbA1c was more prevalent among those born SGA (OR = 1.33 [1.06 to 1.66], *p* = 0.013) and less common among individuals born LGA (OR = 0.81 [0.67 to 0.97], *p* = 0.025). Elevated HbA1c was also more common among individuals born preterm than full-term (OR = 1.42 [1.12 to 1.80], *p* = 0.0036). There was no interaction between weight for GA and GA on elevated HbA1c (*p* = 0.341). Higher OR for elevated HbA1c was observed with increasing age and degree of obesity and for female sex as well as with immigrant background. In contrast to IFG, elevated HbA1c was less common in the BORIS cohort compared to the APV cohort (OR = 0.60 [0.52 to 0.69], *p* < 0.001).

**Table 2:** ORs, 95% CIs, and *p*-values from mutually adjusted logistic regression.

	Hypertensive BP *n* = 38,553		IFG *n* = 25,200		Elevated HbA1c *n* = 9,247		Elevated ALT *n* = 21,297		Elevated total cholesterol *n* = 28,218		Elevated LDL *n* = 26,344		Low HDL *n* = 26,011		Elevated triglycerides *n* = 28,004	
	**OR (95% CI)**	**p**	**OR (95% CI)**	**p**	**OR (95% CI)**	**p**	**OR (95% CI)**	**p**	**OR (95% CI)**	**p**	**OR (95% CI)**	**p**	**OR (95% CI)**	**p**	**OR (95% CI)**	**p**
**LGA vs. AGA**	0.88 (0.83–0.94)	<0.001	0.93 (0.74–1.16)	0.512	0.81 (0.67–0.97)	0.025	0.88 (0.81–0.94)	<0.001	0.96 (0.88–1.06)	0.444	0.99 (0.90–1.08)	0.785	0.97 (0.90–1.05)	0.428	0.90 (0.83–0.97)	0.004
**SGA vs. AGA**	1.20 (1.12–1.29)	<0.001	0.92 (0.67–1.25)	0.587	1.33 (1.06–1.66)	0.013	1.21 (1.10–1.33)	<0.001	1.06 (0.94–1.18)	0.346	1.08 (0.97–1.21)	0.170	1.19 (1.09–1.31)	<0.001	1.08 (0.99–1.18)	0.085
**Male vs. Female**	0.93 (0.89–0.97)	0.002	1.07 (0.90–1.27)	0.446	0.87 (0.76–0.99)	0.039	1.78 (1.68–1.88)	<0.001	1.09 (1.02–1.17)	0.014	1.09 (1.01–1.16)	0.018	1.09 (1.03–1.15)	0.005	0.94 (0.89–0.997)	0.040
**11–14y vs. <11y**	1.36 (1.28–1.43)	<0.001	2.08 (1.66–2.61)	<0.001	1.57 (1.33–1.85)	<0.001	1.53 (1.43–1.64)	<0.001	0.77 (0.71–0.83)	<0.001	0.85 (0.78–0.92)	<0.001	1.88 (1.75–2.03)	<0.001	0.79 (0.74–0.84)	<0.001
**≥14y vs. <11y**	1.41 (1.33–1.49)	<0.001	2.59 (2.07–3.24)	<0.001	1.70 (1.43–2.02)	<0.001	1.84 (1.71–1.98)	<0.001	0.80 (0.73–0.87)	<0.001	0.92 (0.85–1.01)	0.075	2.81 (2.60–3.02)	<0.001	0.86 (0.80–0.92)	<0.001
**Ow vs. Ob I**	0.56 (0.52–0.61)	<0.001	1.20 (0.92–1.58)	0.183	0.86 (0.68–1.09)	ns	0.56 (0.51–0.61)	<0.001	0.95 (0.86–1.05)	0.288	0.89 (0.80–0.99)	0.028	0.65 (0.59–0.72)	<0.001	0.80 (0.73–0.87)	<0.001
**Ob II vs. Ob I**	1.60 (1.51–1.69)	<0.001	0.96 (0.77–1.20)	0.726	1.32 (1.12–1.56)	<0.001	1.60 (1.50–1.72)	<0.001	0.88 (0.80–0.95)	0.002	0.97 (0.89–1.06)	0.511	1.48 (1.38–1.59)	<0.001	1.19 (1.12–1.28)	<0.001
**Ob III vs. Ob I**	2.38 (2.23–2.54)	<0.001	1.73 (1.37–2.19)	<0.001	1.83 (1.51–2.21)	<0.001	2.30 (2.11–2.51)	<0.001	0.81 (0.72–0.90)	<0.001	0.97 (0.89–1.08)	0.611	1.97 (1.81–2.14)	<0.001	1.37 (1.26–1.48)	<0.001
**Immigrant vs. Non-immigrant**	1.09 (1.03–1.15)	0.002	1.16 (0.95–1.42)	0.154	1.25 (1.08–1.45)	0.002	0.95 (0.89–1.02)	0.135	0.94 (0.86–1.02)	0.153	0.92 (0.84–0.999)	0.047	1.03 (0.96–1.10)	0.453	1.00 (0.93–1.07)	0.935
**BORIS vs. APV**	0.39 (0.37–0.41)	<0.001	3.54 (2.96–4.25)	<0.001	0.60 (0.52–0.69)	<0.001	0.79 (0.75–0.85)	<0.001	1.03 (0.95–1.12)	0.438	1.17 (1.08–1.26)	<0.001	1.35 (1.27–1.45)	<0.001	0.92 (0.86–0.98)	0.009

Unadjusted OR for birth weight for gestational age are found in S2 File.

Birth weight status according to Voigt (German reference for birth weight) [23].

Obesity classification according to IOTF [24,25].

Hypertensive BP [26]: Any BP ≥95 percentile for age, sex, and height.

Impaired fasting glucose [29]: ≥6.1 mmol/l respectively 110 mg/dl.

Elevated HbA1c [30]: ≥39 mmol/mol respectively 5.7%.

Elevated ALT [28]: ≥24 U/l respectively 0.4 μkat/l.

Lipids [27]: high cholesterol ≥200 mg/dl; high LDL ≥130 mg/dl; low HDL <40 mg/dl; high triglycerides ≥130 mg/dl (10–19 years) or ≥100 mg/dl (0–9 years).

ALT, alanine aminotransferase; AGA, appropriate for gestational age; BP, blood pressure; HbA1c, glycated hemoglobin; HDL, high density lipoprotein; IOTF, International Obesity Task Force; LDL, low density lipoprotein; LGA, large for gestational age; Ob I, obesity class I; Ob II, obesity class II; Ob III, obesity class III; Ow, overweight; SGA, small for gestational age.

### Blood lipids

Total cholesterol was available in 66.0% (*n* = 28,218), LDL 61.6% (*n* = 26,344), HDL 60.8% (*n* = 26,011), and triglycerides 65.5% (*n* = 28,004) of all individuals. For each respective lipid, derangements were present in 13.6% (*n* = 3,837), 14.5% (*n* = 3,829), 24.8% (*n* = 6,441), and 24.5% (*n* = 6,863).

Birth weight for GA did not affect the risk of elevated total cholesterol nor elevated LDL. However, low HDL was more common among individuals born SGA (OR = 1.19 [1.09 to 1.31], *p* < 0.001) and elevated triglycerides were less common among children born LGA (OR = 0.90 [0.83 to 0.97], *p* = 0.004). No association between preterm birth and any lipid derangements was observed. We found a significant interaction between SGA and preterm birth on elevated LDL (*p* = 0.026), but not on the other lipid components. In the stratified analysis, preterm SGA was associated with higher odds of elevated LDL (OR = 1.50 [1.04 to 2.18], *p* = 0.031) compared to preterm AGA. Such association was not found among full-term SGA (with full-term AGA as comparison), OR = 1.02 [0.91 to 1.15], *p* = 0.46.

### Elevated alanine aminotransferases

ALT was available in 49.8% (*n* = 21,297) and the overall prevalence of elevated ALT was 50.5% (*n* = 10,757). Individuals born SGA were more likely to have elevated ALT (OR = 1.21 [1.10 to 1.33], *p* < 0.001), whereas being born LGA was associated with a lower likelihood (OR = 0.88 [0.81 to 0.94], *p* < 0.001). Preterm birth was not associated with increased risk for elevated ALT (OR = 1.11 [0.99 to 1.23], *p* = 0.059). Significant interaction between GA and weight for gestational age on elevated ALT was detected (*p* < 0.001). The effect of SGA and LGA on elevated ALT became even more pronounced among full-term individuals, while the association was omitted for those born preterm, Fig 2.

### Interaction analyses

We observed an interaction between SGA and weight categories on elevated ALT (*p* = 0.0285), but not on the other outcomes. Neither interaction between SGA and sex nor age on each outcome was found (all *p* > 0.05). Analyses stratified for sex, age group, obesity category, and obesity register are provided in S2 File.

## Discussion

Our study showed that SGA in children and adolescents with overweight or obesity was associated with cardiometabolic risk factors, including increased odds for hypertensive BP, elevated HbA1c, elevated ALT, and low HDL. On the contrary, LGA was associated with lower odds for hypertensive BP, elevated HbA1c, and elevated ALT. Preterm birth modified some of the associations between SGA and cardiometabolic risk factors in children and adolescents with overweight/obesity by increasing the odds for hypertensive BP and by diminishing the odds for elevated ALT.

We found that SGA was associated with increased risk for hypertensive BP in children with overweight or obesity, both in children born preterm and full-term. In line with our finding, 2 hospital-based studies in Germany and Romania of children with overweight found a higher prevalence of hypertension in children born SGA, but the studies included only children born full-term [16,18]. In contrast, results in the general population showed different directions, partly depending on the age when BP was measured. Most studies in adolescents (i.e., 12 years of age or above) found an inverse association between birth weight and BP independently of current children’s weight [8,9], while studies in younger children reported no association [7–9,31]. The independent effect of birth weight on BP seems to be apparent from adolescence [8,9] and persist until adulthood [8,32]. Interestingly, in our study, the positive association between SGA and hypertensive BP was present even in children aged below 11 years (S2 File). This finding implies that the effect of SGA on hypertensive BP might manifest at an earlier age among children with obesity, independent of the degree of obesity.

SGA might affect BP components in children with obesity in a different extent than in the general population. Our study found that SGA was independently associated with increased risk for systolic and diastolic hypertensive BP. Similarly, a German study also found higher mean systolic and diastolic BP in children with excess adiposity born SGA than their counterparts born AGA [16]. On the other hand, studies in the general population indicated that birth weight is positively associated with systolic but not with diastolic BP, both in childhood and adulthood [9,32].

While SGA was an independent risk for hypertensive BP in this study, preterm and SGA seems to have a slightly higher likelihood for hypertensive BP compared to full-term and SGA. A similar finding was shown in a population-based study of Swedish men conscripted for military service with median age of 18 years [14]. This implies that prematurity and SGA may contribute to the development of hypertension through different mechanisms. Increased catecholamine levels, which might contribute to hypertension later in life, seem to be more pronounced in prepubertal children born preterm than those born SGA when compared to full-term AGA [33]. In addition, although prematurity and SGA induce changes in the vascular physiology, their contributions are not easily compared and likely to vary over time [34].

Limited studies in children with overweight or obesity have indicated an association between fetal growth restriction and pediatric non-alcoholic fatty liver disease (NAFLD). However, those studies did not distinguish the independent effect of SGA and preterm birth since GA data was not available [35] or only including children born full-term [16]. In the present study, we have used elevated ALT as a marker for NAFLD and further demonstrated that the association between SGA and NAFLD differs by GA; SGA was associated with an increased risk for NAFLD among individuals with overweight or obesity born full-term, but not among those born preterm. Moreover, studies in children with obesity and biopsy-proven NAFLD showed that SGA status increased the risk for disease progression [35,36]. In contrast, population-based studies indicate contradictive results of the association between SGA and NAFLD. A recent review suggests unclear impact of SGA on NAFLD in children [37]. Epidemiological studies in adults found no association of birth weight and NAFLD, using ALT as a proxy [38,39]. When NAFLD was diagnosed by ultrasound, SGA and preterm birth increased the risk for adult fatty liver in separate models [40].

The link between SGA and NAFLD still needs further investigation. Due to in utero programming, individuals born SGA have been suggested to be prone to rapid catch-up growth and insulin resistance, which contribute to NAFLD and other metabolic diseases [37,41]. However, rapid catch-up growth and insulin resistance could not fully explain the association between SGA and NAFLD as the present study did not observe any major difference in insulin resistance between children born SGA and AGA. Similarly, the associations of SGA with NAFLD [12] and severity of steatosis [36] were independent of insulin resistance and BMI SDS according to previous studies. Adverse intrauterine environment might trigger epigenetic regulation and impaired liver growth, to some extent independently of insulin resistance and degree of obesity [42].

In the general pediatric population, SGA is associated with higher degree of insulin resistance, but results vary between studies, depending somewhat on the biomarkers used to measure insulin resistance [10,11,43]. Only few studies have investigated the association between GA and HbA1c in children [31,43]. The present study found that SGA was associated with elevated HbA1c. This finding is in line with a population-based study in Canada where most participants had normal weight [31]. On the other hand, we observed no association between SGA and IFG. This finding is in accordance with other studies in children with overweight or obesity [16,17,19]. However, the previous studies have shown that SGA status in children with overweight or obesity was associated with increased risk for impaired glucose tolerance (IGT), a third category of intermediate hyperglycemia [16,17,19]. As the prediabetic states IFG and IGT are mediated by different mechanisms, where IFG is derived from hepatic IR, and IGT primarily is caused by peripheral IR [44], it is plausible that SGA affects later peripheral IR but not hepatic IR.

Although fetal growth restriction seems to impact glucose metabolism in children, SGA and preterm birth might affect different aspects of diabetes pathophysiology distinctively according to some large population-based studies. SGA and/or preterm birth have been shown as risk factors for type 2 diabetes in children [10,45]. Intriguingly, while preterm birth has been demonstrated as a risk factor for type 1 diabetes [45], SGA seems to have a protective effect on type 1 diabetes [46]. These findings suggest distinct consequences of SGA and preterm birth on glucose metabolism and diabetes pathogenesis.

HDL and triglycerides are 2 lipid components included in metabolic syndrome criteria, and derangements in them were present in 25% of our study population. The present study found that SGA was associated with low HDL, but not with elevated triglycerides. In contrast, other studies in children with overweight or obesity have reported the opposite [16,17]. A possible explanation for this discrepancy was that the previous study did not adjust the result for weight status [17], whereas BMI SDS was controlled in the present study. In addition, it is possible that in the pediatric population with obesity, the metabolic impact of SGA status is not substantial enough to affect triglyceride levels. Even though SGA has been associated with dyslipidemia in the general pediatric population [10], most large studies in children did not find any association between SGA and lipid profile, particularly when the results were adjusted for weight status [7,13,31]. Hence, the effect of adiposity on lipid profile might overshadow the effect of SGA.

Although SGA was associated with adverse cardiometabolic profile in children with overweight or obesity, the effect of SGA was very modest compared to the effect of obesity severity. For example, odds for hypertensive BP were 1.2 times higher among children born SGA than those born AGA, while the odds were 2.4 times higher among children with obesity class III than class I obesity after adjustment for SGA status. This finding suggests that adiposity, rather than perinatal factors, plays a more important role in cardiometabolic profile in children with overweight or obesity. Moreover, our study also indicated that, compared to children with obesity, children with overweight had lower risk for adverse cardiometabolic profile independently of perinatal factors. These results emphasize the importance of weight management, which might outweigh the unfavorable impact of SGA in children with obesity.

The mechanism linking fetal growth restriction on later cardiometabolic diseases has been attempted to be explained by the thrifty phenotype hypothesis, whereby in utero malnutrition induces fetal programming which in long-term leads to adverse cardiometabolic profile. Adiposity has been argued to be one of the mediators of this mechanism. However, the present study found that the unfavorable effect of SGA was independent of obesity severity and thereby questions adiposity as mediator. Moreover, children born SGA were less likely to have severe obesity (Table 1).

According to the developmental origins concept, people with a nutritional mismatch between prenatal and postnatal environments, i.e., being born small and growing big in childhood, would face the highest risk of later cardiometabolic disease [47]. In accordance with this concept, a better pre-postnatal match in living conditions may have contributed to the significantly lower odds for hypertensive BP and elevated HbA1c found in children with obesity born LGA. However, such an association may change over time and potential cardiometabolic disadvantages of being born LGA may not be possible to detect in childhood [31].

Other studies have found individuals born SGA to be underrepresented among the pediatric obesity population [19,20]; however, our data did not support that. In a Swedish study that investigated 3.8 million births during a similar period, SGA was present in 7.8% [48], while our cohorts combined demonstrated an SGA prevalence of 12.0% using the same reference and cut-off for SGA. However, since normal weight controls were not included, our study was not designed to disentangle whether SGA increases the risk for obesity in pediatric years. Moreover, our study was not designed to evaluate whether combination of SGA and obesity is worse than the independent effect of SGA on cardiometabolic risks in general pediatric population.

The large sample size in this study enabled us to investigate potential differences in GA, sex, and age at outcome measures regarding the association between birth size and cardiometabolic profile in children with obesity. Moreover, to our knowledge, no prior studies in children with overweight or obesity have used multiple national cohorts to assess the association between perinatal factors and cardiometabolic profiles. Data from national cohorts improved the external validity of our findings. There are, however, limitations of the current study that may impact our results. Despite our large sample of children and adolescents with overweight or obesity, some observed associations in stratified analyses resulted in large confidence limits and were not statistically significant (e.g., the double odds for elevated HbA1c among children with obesity born preterm and SGA), possibly due to the lack of power. When interpreting the stratified analyses, one should bear in mind that no interaction between weight for GA and the stratified variable were present in most analyses; hence, we could not rule out the influence of chance variation in the associations observed in some subgroups. Moreover, some outcome variables had a rather large proportion of missing data, e.g., HbA1c data was available in 21% of the study population. Reasons for occasionally missing data of the investigated outcome variables are unknown; hence, there might be potential selection bias that could distort the magnitude of the association. Moreover, since our data sources were obesity registers in European countries, the generalizability of the findings might be limited to children with overweight or obesity with similar circumstances as the present study. Further, we did not have data on the causes for deviating birth weight and GA, e.g., substance use, intrauterine infection, placental insufficiency, and maternal stress. Further, attributes during pregnancy (e.g., gestational diabetes, smoking, and weight gain), hereditary factors, and socioeconomic position were not available in the present study. Also, postnatal characteristics, such as catch-up weight and breast feeding were not documented. It is possible that these factors may affect the investigated associations, by e.g., altered epigenetic profile. Nevertheless, it has been shown that postnatal weight gain leads to cardiometabolic risk in childhood through its effect on childhood adiposity [7]. Since our study population was children with excess adiposity, the lack of postnatal weight data was unlikely to change the direction of the association reported in this study. Future research including postnatal characteristics and maternal attributes during pregnancy would allow better understanding of mechanism linking SGA and cardiometabolic risk in excess adiposity environment.

In conclusions, among children and adolescents with overweight/obesity, individuals born SGA are more likely to possess cardiometabolic risk factors compared to their counterparts born AGA. Target screening and treatment of obesity-related comorbidities should therefore be considered in this high-risk group of individuals.

## Supporting information

S1 File STROBE checklist. (DOCX)

S2 File Additional information and analyses. Description of the obesity registers. S1 Table. Characteristics of individuals stratified for obesity register. S2 Table. Unadjusted odds ratios. S3 Table. Unadjusted odds ratios stratified for gestational age. S4 Table. Odds ratios based on Maršál reference. S1 Fig. Odds ratios stratified for gestational age. S2 Fig. Odds ratios for systolic and diastolic blood pressure. S5 Table. Odds ratios stratified for sex. S6 Table. Odds ratios stratified for age group. S7 Table. Odds ratios stratified for weight status. S8 Table. Odds ratios stratified for obesity register. References.
(PDF)

S3 File Project description. (DOCX)

## Abbreviations

AGA: appropriate for gestational age
ALT: alanine aminotransferase
BMI SDS: body mass index standard deviation score
BP: blood pressure
CI: confidence interval
GA: gestational age
HDL: high-density lipoprotein
IFG: impaired fasting glycemia
IGT: impaired glucose tolerance
LDL: low-density lipoprotein
LGA: large for gestational age
NAFLD: non-alcoholic fatty liver disease
OR: odds ratio
SGA: small for gestational age
