# Supplementary material for: The association between perinatal factors and cardiometabolic risk factors in children and adolescents with overweight or obesity: A retrospective two-cohort study
Source: PLoS Med. 2023 Jan 13;20(1):e1004165. doi: 10.1371/journal.pmed.1004165 (PMC9886302; doi:10.1371/journal.pmed.1004165)
Supplement: S1 File — (DOCX) [file pmed.1004165.s001.docx]

STROBE Checklist

|  | Item No | Recommendation | Section/Paragraph |
| --- | --- | --- | --- |
| **Title and abstract** | 1 | (*a*) Indicate the study’s design with a commonly used term in the title or the abstract | Indicated in the title |
|  |  | (*b*) Provide in the abstract an informative and balanced summary of what was done and what was found | Indicated in the abstract |
| Introduction | | | |
| Background/rationale | 2 | Explain the scientific background and rationale for the investigation being reported | Explanations provided in the Background  section |
| Objectives | 3 | State specific objectives, including any prespecified hypotheses | Objectives are stated at the end of the Background section |
| Methods | | | |
| Study design | 4 | Present key elements of study design early in the paper | Presented in the first paragraph of the method section and shown in Figure 1. |
| Setting | 5 | Describe the setting, locations, and relevant dates, including periods of recruitment, exposure, follow-up, and data collection | Described throughout Methods section |
| Participants | 6 | (*a*) Give the eligibility criteria, and the sources and methods of selection of participants. Describe methods of follow-up | Presented in the first and second paragraph of the Methods section. |
|  |  | (*b*) For matched studies, give matching criteria and number of exposed and unexposed | N/A |
| Variables | 7 | Clearly define all outcomes, exposures, predictors, potential confounders, and effect modifiers. Give diagnostic criteria, if applicable | Presented in the Methods section under subheading Variables and definitions. |
| Data sources/ measurement | 8* | For each variable of interest, give sources of data and details of methods of assessment (measurement). Describe comparability of assessment methods if there is more than one group | Described in the Methods section under each subsection. |
| Bias | 9 | Describe any efforts to address potential sources of bias | Described in the first paragraph of the Method section and under subheading Statistical Analyses |
| Study size | 10 | Explain how the study size was arrived at | Described in the first paragraph of the method and presented in Figure 1. |
| Quantitative variables | 11 | Explain how quantitative variables were handled in the analyses. If applicable, describe which groupings were chosen and why | Described in the methods under subheadings “Variables and definitions” |
| Statistical methods | 12 | (*a*) Describe all statistical methods, including those used to control for confounding | Described in the methods, subheading “Statistical analyses”, paragraph 2 |
|  |  |  |  |
|  |  | (*b*) Describe any methods used to examine subgroups and interactions | Described in the methods, subheading “Statistical analyses”, paragraph 2 |
|  |  |  |  |
|  |  | (*c*) Explain how missing data were addressed | Described in the methods, subheading “Statistical analyses”, paragraph 1 |
|  |  |  |  |
|  |  | (*d*) If applicable, explain how loss to follow-up was addressed | Not applicable |
|  |  |  |  |
|  |  | (*e*) Describe any sensitivity analyses | Not applicable |
| Results | | |  |
| Participants | 13* | (a) Report numbers of individuals at each stage of study—eg numbers potentially eligible, examined for eligibility, confirmed eligible, included in the study, completing follow-up, and analysed | Reported in Results section for each analysis and shown in Figure 1 |
|  |  | (b) Give reasons for non-participation at each stage | Included as Figure 1 |
|  |  | (c) Consider use of a flow diagram | Included as Figure 1. |
| Descriptive data | 14* | (a) Give characteristics of study participants (eg demographic, clinical, social) and information on exposures and potential confounders | Reported in the Results section paragraph 1 and 2 and Table 1 |
|  |  | (b) Indicate number of participants with missing data for each variable of interest | Reported in the Results section under subheading “Hypertensive blood pressure”, “Glucose metabolism”, “Blood lipids”, “Elevated alanine aminotransferases” |
|  |  | (c) Summarise follow-up time (eg, average and total amount) | Reported in the Results section paragraph 2 |
| Outcome data | 15* | Report numbers of outcome events or summary measures over time | Reported in the Results section under subheading “Hypertensive blood pressure”, “Glucose metabolism”, “Blood lipids”, “Elevated alanine aminotransferases” and Table 1 |

| Main results | 16 | (*a*) Give unadjusted estimates and, if applicable, confounder-adjusted estimates and their precision (eg, 95% confidence interval). Make clear which confounders were adjusted for and why they were included | Reported in Table 2 and Supplementary File 2 |
| --- | --- | --- | --- |
|  |  | (*b*) Report category boundaries when continuous variables were categorized | Reported in in the methods, subheading “Statistical analyses”, paragraph 1 and footnotes of each table |
|  |  | (*c*) If relevant, consider translating estimates of relative risk into absolute risk for a meaningful time period | Not applicable |
| Other analyses | 17 | Report other analyses done—eg analyses of subgroups and interactions, and sensitivity analyses | Reported in the Results section under subheading “Hypertensive blood pressure”, “Glucose metabolism”, “Blood lipids”, “Elevated alanine aminotransferases”, “Stratified analyses”, Figure 2, and Supplementary File 2 |
| Discussion | | | |
| Key results | 18 | Summarise key results with reference to study objectives | Described in the Discussion section paragraph 1 |
| Limitations | 19 | Discuss limitations of the study, taking into account sources of potential bias or imprecision. Discuss both direction and magnitude of any potential bias | Described in the Discussion section paragraph 14 (one paragraph before conclusion) |
| Interpretation | 20 | Give a cautious overall interpretation of results considering objectives, limitations, multiplicity of analyses, results from similar studies, and other relevant evidence | Described in the Discussion section paragraph 14 (one paragraph before conclusion) |
| Generalisability | 21 | Discuss the generalisability (external validity) of the study results | Described in the Discussion section paragraph 14 (one paragraph before conclusion) |
| Other information | | | |
| Funding | 22 | Give the source of funding and the role of the funders for the present study and, if applicable, for the original study on which the present article is based | Funding statement is reported separately |

*Give information separately for exposed and unexposed groups.

**Note:** An Explanation and Elaboration article discusses each checklist item and gives methodological background and published examples of transparent reporting. The STROBE checklist is best used in conjunction with this article (freely available on the Web sites of PLoS Medicine at http://www.plosmedicine.org/, Annals of Internal Medicine at http://www.annals.org/, and Epidemiology at http://www.epidem.com/). Information on the STROBE Initiative is available at http://www.strobe-statement.org.
