# Supplementary material for: The association between perinatal factors and cardiometabolic risk factors in children and adolescents with overweight or obesity: A retrospective two-cohort study
Source: PLoS Med. 2023 Jan 13;20(1):e1004165. doi: 10.1371/journal.pmed.1004165 (PMC9886302; doi:10.1371/journal.pmed.1004165)
Supplement: S2 File — Description of the obesity registers. S1 Table. Characteristics of individuals stratified for obesity register. S2 Table. Unadjusted odds ratios. S3 Table. Unadjusted odds ratios stratified for gestational age. S4 Table. Odds ratios based on Maršál reference. S1 Fig. Odds ratios stratified for gestational age. S2 Fig. Odds ratios for systolic and diastolic blood pressure. S5 Table. Odds ratios stratified for sex. S6 Table. Odds ratios stratified for age group. S7 Table. Odds ratios stratified for weight status. S8 Table. Odds ratios stratified for obesity register. References. (PDF) [file pmed.1004165.s002.pdf]

Supplementary File 2

This appendix formed part of the original submission and has been peer reviewed.

Contents

Description of the obesity registers 2

Supplementary Table 1. Characteristics of individuals stratified for obesity register 3

Supplementary Table 2. Unadjusted odds ratios 4

Supplementary Table 3. Unadjusted odds ratios stratified for gestational age 5

Supplementary Table 4. Odds ratios (OR) based on Maršál reference 6

Supplementary Figure 1. Odds ratios (OR) stratified for gestational age 7

Supplementary Figure 2. Odds ratios (OR) for systolic and diastolic blood pressure. 8

Supplementary Table 5. Odds ratios (OR) stratified for sex 9

Supplementary Table 6. Odds ratios (OR) stratified for age group 10

Supplementary Table 7. Odds ratios (OR) stratified for weight status 11

Supplementary Table 8. Odds ratios (OR) stratified for obesity register 12

References 13

Description of the obesity registers

APV

The Adiposity Patients Registry (APV) is a standardized, multicenter registry for ongoing documentation of anthropometric and metabolic data in people with overweight or obesity, mainly of pediatric and young adult ages. Specialized centers in obesity (inpatient rehabilitation or outpatient) from Germany, Austria and Switzerland are contributing anonymized data that were transmitted to Ulm, Germany, and aggregated into a cumulative database for clinical research and quality assurance [1,2]. Plausibility checks and correction of inconsistent data are performed semi-annually. Until December 2020, 228 centers participated in this APV initiative. Some people documented in the registry start a lifestyle intervention (LI) program for obesity and are followed-up. Besides demographics, anthropometrics and clinical parameters at the start of the LI program and during follow-up, birth characteristics as well as other relevant family information (like parental BMI, family situation, etc.) are collected in a subgroup of people. 157 centers contributed data to the present analysis.

The Swedish Childhood Obesity Treatment Register (BORIS)

The Swedish Childhood Obesity Treatment Register (BORIS) is a prospective register of children and adolescents undergoing obesity treatment [3]. Guidelines for obesity treatment in Sweden include that treatment should be initiated at an early age and before a severe obesity is manifested. Treatment focus on lifestyle modification to reduce the degree of obesity by improving dietary habits and increasing physical activity. No pharmacological treatment is currently available. All health care, including treatment of obesity, is free of charge for children and adolescents up to 18 years of age. Generally, treatment is aimed to be tailored towards the families' needs and abilities to adhere to specific programs. Treatment may therefore be delivered differently.

According to Swedish regulations, families were informed in writing and verbally about data collection in BORIS. Post an opt-out approval (possibility to choose not to participate) by parents/guardians, data of the children's weight and height were recorded by the local healthcare provider during treatment visits.

For individuals in BORIS, birth data was collected from the Swedish medical birth register. In Sweden all residents are assigned a unique personal identification number, which was used by the governmental agency the National Board of Health and Welfare to link the two registers on an individual level. The Swedish Medical Birth Register was founded in 1973 and includes data on almost all deliveries in Sweden, since it is compulsory for all health care providers to report to the register.

Supplementary Table 1. Characteristics of individuals stratified for obesity register.

	APV (Germany/Austria/Switzerland)				BORIS (Sweden)				p-value	Total			
	**n**	**Median**	**Q1**	**Q3**	**n**	**Median**	**Q1**	**Q3**		**n**	**Median**	**Q1**	**Q3**
Age (years)	30 327	12.3	10.1	14.2	12 433	10.4	7.8	13.1	<0.0001	42 760	11.8	9.4	14.0
BMI (kg/m^2)	30 327	29.1	25.7	33.2	12 433	27.0	23.7	31.2	<0.001	42 760	28.5	25.1	32.7
BMI SDS	30 327	2.7	2.4	3.1	12 433	2.8	2.5	3.2	<0.0001	42 760	2.74	2.40	3.11
Gestational age (weeks)	30 327	40.0	38.0	40.0	12 433	40.0	38.0	41.0	0.0007	42 760	40.0	38.0	40.0
Birth weight (gram)	30 327	3 450	3 090	3 800	12 433	3 630	3 275	4 010	<0.0001	42 760	3500	3140	3870
Birth length (cm)	27 843	52.0	50.0	53.0	12 277	50.0	49.0	52.0	<0.0001	40 120	51	50	53
Gestational weight SDS (Voigt et al.[4])	30 327	0.06	-0.72	0.84	12 433	0.44	-0.27	1.20	<0.0001	42 760	0.17	-0.60	0.96
Gestational weight SDS (Maršál et al.[5])	30 327	-0.04	-0.81	0.8	12 433	0.18	-0.53	0.97	<0.0001	42 760	0.04	-0.73	0.85
Maternal age at birth (years)	30 196	25.5	22.2	29.8	12 433	29.9	25.9	33.9	<0.0001	42 629	26.8	22.9	31.3
Systolic BP (mmHg)	28 221	120.0	110.0	130.0	10 332	111.0	105.0	120.0	<0.001	38 553	118	110	127
Systolic BP Z-score[6]	28 221	1.01	0.25	1.83	10 332	0.58	-0.05	1.25	<0.0001	38 553	0.88	0.16	1.68
Diastolic BP (mmHg)	28 221	70.0	64.0	80.0	10 332	66.0	60.0	72.0	<0.001	38 553	70	62	79
Diastolic BP Z-score[6]	28 221	0.69	0.12	1.34	10 332	0.38	-0.12	0.89	<0.0001	38 553	0.59	0.02	1.24
Fasting glucose (mmol/l)	17 704	4.7	4.4	5.1	7 496	5.1	4.8	5.4	<0.0001	25 200	4.8	4.4	5.2
HbA1c/IFCC/(mmol/mol)	3 440	34.4	32.2	36.6	5 807	34.0	32.0	36.4	<0.0001	9 247	34.4	32.2	36.6
Total cholesterol (mmol/l)	21 053	4.2	3.6	4.8	7 165	4.3	3.8	4.8	<0.0001	28 218	4.2	3.7	4.8
Triglycerides (mmol/l)	20 849	1.0	0.7	1.4	7 155	0.9	0.6	1.3	<0.0001	28 004	0.9	0.7	1.4
LDL cholesterol (mmol/l)	19 149	2.5	2.0	3.0	7 195	2.6	2.2	3.1	<0.0001	26 344	2.5	2.1	3.1
HDL cholesterol (mmol/l)	18 821	1.2	1.0	1.4	7 190	1.2	1.0	1.4	0.0118	26 011	1.2	1.0	1.4
ALT (µkat/l)	13 522	0.4	0.3	0.6	7 775	0.4	0.3	0.5	<0.0001	21 297	0.4	0.3	0.6

ALT, alanine aminotransferase; AGA, appropriate for gestational age; BMI, body mass index; BP, blood pressure; HbA1c, glycated hemoglobin; HDL, high density lipoprotein; LDL, low density lipoprotein; LGA, large for gestational age; OR, odds ratio; SDS, standard deviation score; SGA, small for gestational age.

Supplementary Table 2. Unadjusted odds ratios (OR) from logistic regression. Results are presented with and 95% confidence intervals (CI) and p-values. The corresponding adjusted values are presented in Table 2 in the article (German reference) and Supplementary Table 4 (Swedish reference).

	Hypertensive blood pressure n = 38 553		Impaired fasting glycemia n = 25 200		Elevated HbA1c n = 9 247		Elevated ALT n = 21 297		Elevated total cholesterol n = 28 218		Elevated LDL n = 26 344		Low HDL n = 26 011		Elevated Triglycerides n = 28 004	
	**OR (CI)**	**p**	**OR (CI)**	**p**	**OR (CI)**	**p**	**OR (CI)**	**p**	**OR (CI)**	**p**	**OR (CI)**	**p**	**OR (CI)**	**p**	**OR (CI)**	**p**
**German reference**[4]																
**LGA vs. AGA**	0.89 (0.83 – 0.94)	<0.001	1.05 (0.83 – 1.31)	0.667	0.77 (0.64 – 0.92)	0.004	0.89 (0.83 – 0.96)	0.002	0.97 (0.88 – 1.07)	0.496	1.02 (0.93 – 1.11)	0.748	1.02 (0.95 – 1.10)	0.538	0.93 (0.86 – 0.99)	0.041
**SGA vs. AGA**	1.28 (1.20 – 1.38)	<0.001	0.81 (0.58 – 1.11)	0.191	1.46 (1.16 – 1.82)	<0.001	1.25 (1.14 – 1.37)	<0.001	1.04 (0.93 – 1.17)	0.477	1.06 (0.94 – 1.19)	0.323	1.18 (1.08 – 1.30)	<0.001	1.07 (0.98 – 1.17)	0.117
**Swedish reference**[5]																
**LGA vs. AGA**	0.96 (0.91 – 1.02)	0.241	1.00 (0.78 – 1.26)	0.970	0.68 (0.56 – 0.83)	<0.001	0.96 (0.89 – 1.04)	0.288	0.93 (0.85 – 1.03)	0.151	0.99 (0.90 – 1.09)	0.817	1.03 (0.96 – 1.12)	0.409	0.97 (0.90 – 1.04)	0.366
**SGA vs. AGA**	1.29 (1.21 – 1.38)	<0.001	0.81 (0.60 – 1.08)	0.150	1.34 (1.10 – 1.63)	0.003	1.24 (1.14 – 1.35)	<0.001	1.02 (0.91 – 1.13)	0.765	1.06 (0.95 – 1.18)	0.313	1.18 (1.08 – 1.29)	<0.001	1.09 (0.99 – 1.18)	0.051

Hypertensive blood pressure [6]: Any blood pressure ≥95 percentile for age, sex, and height

Impaired fasting glycemia [7]: ≥6.1 mmol/l respectively 110 mg/dl

Elevated HbA1c [8]: ≥39 mmol/mol respectively 5.7%

Elevated ALT [9]: ≥24 U/l respectively 0.4 µkat/l

Altered lipids [10]: Cholesterol ≥200 mg/dl; LDL ≥130 mg/dl; HDL <40 mg/dl; TG ≥130 mg/dl (10 – 19 years) or ≥100 mg/dl (0 – 9 years)
ALT, alanine aminotransferase; AGA, appropriate for gestational age; HbA1c, glycated hemoglobin; HDL, high density lipoprotein; LDL, low density lipoprotein; LGA, large for gestational age; OR, odds ratio; SGA, small for gestational age

Supplementary Table 3. Unadjusted odds ratios (OR) stratified for gestational age from logistic regression. Results are presented with and 95% confidence intervals (CI) and p-values. The corresponding adjusted values are presented in Figure 2 and Supplementary Figure 1.

		**Hypertensive blood pressure n = 38 553**		**Impaired fasting glycemia n = 25 200**		**Elevated HbA1c n = 9 247**		**Elevated ALT n = 21 297**		**Elevated total cholesterol n = 28 218**		**Elevated LDL n = 26 344**		**Low HDL n = 26 011**		**Elevated Triglycerides n = 28 004**	
**Strata**		**OR (CI)**	**p**	**OR (CI)**	**p**	**OR (CI)**	**p**	**OR (CI)**	**p**	**OR (CI)**	**p**	**OR (CI)**	**p**	**OR (CI)**	**p**	**OR (CI)**	**p**
	**German reference**[4]																
**Preterm**	**LGA vs. AGA**	0.90 (0.75 – 1.09)	0.279	1.06 (0.49 – 2.31)	0.885	0.76 (0.41 – 1.41)	0.379	1.19 (0.94 – 1.50)	0.144	0.98 (0.73 – 1.31)	0.887	0.76 (0.56 – 1.04)	0.206	0.99 (0.77 – 1.27)	0.907	0.81 (0.64 – 1.02)	0.067
**Full term**	**LGA vs. AGA**	0.88 (0.82 – 0.93)	<0.001	1.06 (0.83 – 1.33)	0.645	0.77 (0.63 – 0.93)	<0.001	0.86 (0.80 – 0.93)	<0.001	0.97 (0.88 – 1.07)	0.530	1.05 (0.95 – 1.15)	0.360	1.03 (0.95 – 1.12)	0.464	0.94 (0.87 – 1.01)	0.093
**Preterm**	**SGA vs. AGA**	1.34 (1.05 – 1.71)	0.018	1.07 (0.37 – 3.13)	0.903	2.33 (1.14 – 4.77)	0.021	0.79 (0.57 – 1.09)	0.149	1.50 (1.03 – 2.18)	0.033	1.46 (1.01 – 2.11)	0.044	0.91 (0.64 – 1.30)	0.602	0.76 (0.54 – 1.06)	0.105
**Full term**	**SGA vs. AGA**	1.28 (1.19 – 1.37)	<0.001	0.80 (0.58 – 1.10)	0.169	1.40 (1.11 – 1.77)	0.005	1.30 (1.18 – 1.43)	<0.001	1.01 (0.90 – 1.13)	0.897	1.03 (0.91 – 1.15)	0.686	1.21 (1.10 – 1.33)	<0.001	1.10 (1.01 – 1.21)	0.037
	**Swedish reference**[5]																
**Preterm**	**LGA vs. AGA**	1.01 (0.85 – 1.21)	0.892	0.97 (0.45 – 2.11)	0.945	0.82 (0.44 – 1.51)	0.523	1.26 (1.004 – 1.59)	0.046	0.97 (0.73 – 1.29)	0.817	0.79 (0.58 – 1.07)	0.274	1.06 (0.83 – 1.35)	0.881	0.82 (0.66 – 1.03)	0.088
**Full term**	**LGA vs. AGA**	0.95 (0.89 – 1.01)	0.113	1.01 (0.79 – 1.29)	0.928	0.78 (0.64 – 0.96)	0.020	0.92 (0.85 – 0.99)	0.029	0.93 (0.84 – 1.03)	0.167	1.02 (0.92 – 1.13)	0.996	1.04 (0.95 – 1.12)	0.415	0.98 (0.90 – 1.06)	0.559
**Preterm**	**SGA vs. AGA**	1.47 (1.21 – 1.80)	<0.001	0.97 (0.40 – 2.32)	0.942	2.22 (1.30 – 3.79)	0.004	0.97 (0.75 – 1.27)	0.844	1.26 (0.92 – 1.73)	0.153	1.36 (0.99 – 1.85)	0.125	1.04 (0.79 – 1.37)	0.958	0.89 (0.68 – 1.15)	0.372
**Full term**	**SGA vs. AGA**	1.26 (1.18 – 1.35)	<0.001	0.80 (0.59 – 1.09)	0.154	1.43 (1.16 – 1.78)	0.001	1.28 (1.17 – 1.40)	<0.001	0.99 (0.88 – 1.11)	0.831	1.01 (0.91 – 1.14)	0.967	1.21 (1.10 – 1.32)	<0.001	1.10 (1.01 – 1.21)	0.031

Hypertensive blood pressure [6]: Any blood pressure ≥95 percentile for age, sex, and height

Impaired fasting glycemia [7]: ≥6.1 mmol/l respectively 110 mg/dl

Elevated HbA1c [8]: ≥39 mmol/mol respectively 5.7%

Elevated ALT [9]: ≥24 U/l respectively 0.4 µkat/l

Altered lipids [10]: Cholesterol ≥200 mg/dl; LDL ≥130 mg/dl; HDL <40 mg/dl; TG ≥130 mg/dl (10 – 19 years) or ≥100 mg/dl (0 – 9 years)
ALT, alanine aminotransferase; AGA, appropriate for gestational age; HbA1c, glycated hemoglobin; HDL, high density lipoprotein; LDL, low density lipoprotein; LGA, large for gestational age; OR, odds ratio; SGA, small for gestational age

Supplementary Table 4. Odds ratios (OR), 95% confidence intervals (CI), and p-values from mutually adjusted logistic regression. Birthweight for gestational age are based on Maršál reference[5] (Swedish). Unadjusted OR for birth weight categories are found in Supplementary Table 2.

	**Hypertensive blood pressure n = 38 553**		**Impaired fasting glycemia n = 25 200**		**Elevated HbA1c n = 9 247**		**Elevated ALT n = 21 297**		**Elevated total cholesterol n = 28 218**		**Elevated LDL n = 26 344**		**Low HDL n = 26 011**		**Elevated Triglycerides n = 28 004**	
	**OR (CI)**	**p**	**OR (CI)**	**p**	**OR (CI)**	**p**	**OR (CI)**	**p**	**OR (CI)**	**p**	**OR (CI)**	**p**	**OR (CI)**	**p**	**OR (CI)**	**p**
**LGA vs. AGA**	0.90 (0.85 – 0.96)	0.001	0.96 (0.76 – 1.22)	0.752	0.79 (0.65 – 0.97)	0.021	0.91 (0.84 – 0.98)	0.021	0.94 (0.85 - 1.03)	0.169	0.98 (0.89 – 1.07)	0.630	0.98 (0.91 – 1.06)	0.615	0.94 (0.87 – 1.01)	0.104
**SGA v. AGA**	1.23 (1.15 – 1.32)	<0.001	0.87 (0.65 – 1.17)	0.354	1.44 (1.18 – 1.76)	<0.001	1.23 (1.12 – 1.34)	<0.001	1.03 (0.93 - 1.15)	0.579	1.08 (0.97 – 1.20)	0.158	1.18 (1.08 – 1.29)	<0.001	1.09 (1.00 – 1.19)	0.040
**Male vs. Female**	0.94 (0.90 – 0.98)	0.004	1.07 (0.90 – 1.27)	0.453	0.88 (0.77 – 1.01)	0.061	1.79 (1.69 – 1.89)	<0.001	1.09 (1.02 - 1.17)	0.012	1.09 (1.02 – 1.17)	0.016	1.09 (1.03 – 1.16)	0.004	0.95 (0.90 – 1.00)	0.054
**11-14y vs. <11y**	1.35 (1.28 – 1.43)	<0.001	2.08 (1.66 – 2.61)	<0.001	1.56 (1.33 – 1.84)	<0.001	1.53 (1.43 – 1.64)	<0.001	0.77 0.71 – 0.83	<0.001	0.85 (0.78 – 0.92)	<0.001	1.88 (1.75 – 2.03)	<0.001	0.79 (0.74 - 0.84)	<0.001
**≥14y vs. <11y**	1.41 (1.33 – 1.49)	<0.001	2.60 (2.08 – 3.25)	<0.001	1.69 (1.42 – 2.01)	<0.001	1.84 (1.71 – 1.98)	<0.001	0.80 (0.73 - 0.87)	<0.001	0.92 (0.85 – 1.01)	0.075	2.81 (2.60 – 3.02)	<0.001	0.86 (0.80 – 0.92)	<0.001
**Ow vs. Ob I**	0.56 (0.52 – 0.61)	<0.001	1.21 (0.92 – 1.58)	0.180	0.86 (0.68 – 1.09)	0.208	0.56 (0.51 – 0.61)	<0.001	0.95 (0.85 - 1.05)	0.284	0.89 (0.80 – 0.99)	0.028	0.65 (0.59 – 0.72)	<0.001	0.80 (0.74 – 0.88)	<0.001
**Ob II vs. Ob I**	1.60 (1.51 – 1.69)	<0.001	0.96 (0.77 – 1.20)	0.714	1.32 (1.12 – 1.56)	0.001	1.60 (1.49 – 1.71)	<0.001	0.88 (0.80 - 0.95)	0.002	0.97 (0.89 – 1.06)	0.517	1.48 (1.38 – 1.59)	<0.001	1.19 (1.12 – 1.28)	<0.001
**Ob III vs. Ob I**	2.38 (2.23 – 2.54)	<0.001	1.73 (1.37 – 2.18)	<0.001	1.83 (1.52 – 2.22)	<0.001	2.29 (2.10 – 2.50)	<0.001	0.81 (0.73 - 0.87)	<0.001	0.97 (0.88 – 1.08)	0.620	1.97 (1.81 – 2.14)	<0.001	1.36 (1.26 – 1.48)	<0.001
**Immigrant vs. Non-immigrant**	1.09 (1.03 – 1.15)	0.002	1.16 (0.95 – 1.42)	0.144	1.25 (1.08 – 1.45)	0.003	0.95 (0.89 – 1.02)	0.146	0.94 (0.86 - 1.02)	0.153	0.92 (0.84 – 0.99)	0.046	1.03 (0.96 – 1.10)	0.455	1.00 (0.93 – 1.07)	0.967
**BORIS vs. APV**	0.38 (0.36 – 0.41)	<0.001	3.53 (2.94 – 4.22)	<0.001	0.59 (0.52 – 0.68)	<0.001	0.79 (0.74 – 0.84)	<0.001	1.03 (0.95 -–1.12)	0.481	1.16 (1.08 – 1.26)	<0.001	1.35 (1.26 – 1.44)	<0.001	0.91 (0.85 – 0.97)	0.005

Obesity classification according to IOTF [11,12]

Hypertensive blood pressure [6]: Any blood pressure ≥95 percentile for age, sex, and height

Impaired fasting glycemia [7]: ≥6.1 mmol/l respectively 110 mg/dl

Elevated HbA1c [8]: ≥39 mmol/mol respectively 5.7%

Elevated ALT [9]: ≥24 U/l respectively 0.4 µkat/l

Altered lipids [10]: Cholesterol ≥200 mg/dl; LDL ≥130 mg/dl; HDL <40 mg/dl; TG ≥130 mg/dl (10 – 19 years) or ≥100 mg/dl (0 – 9 years)

ALT, alanine aminotransferase; AGA, appropriate for gestational age; BP, blood pressure; CI; confidence interval; HbA1c, glycated hemoglobin; HDL, high density lipoprotein; LDL, low density lipoprotein; LGA, large for gestational age; Ob I, obesity class I; Ob II, obesity class II; Ob III, obesity class III; OR, odds ratio; Ow, overweight; SGA, small for gestational age.

	Odds Ratio (95% CI)	p-value
**Hypertensive blood pressure**		
Full term SGA	1.19 (1.11 – 1.28)	<.0001
Full term LGA	0.91 (0.85 – 0.97)	0.0048
Preterm SGA	1.55 (1.26 – 1.91)	<.0001
Preterm LGA	0.86 (0.71 – 1.04)	0.12
**IFG**		
Full term SGA	0.86 (0.63 – 1.17)	0.35
Full term LGA	0.96 (0.75 – 1.23)	0.75
Preterm SGA	1.00 (0.41 – 2.43)	1.00
Preterm LGA	1.11 (0.50 – 2.46)	0.80
**Elevated HbA1c**		
Full term SGA	1.32 (1.06 – 1.65)	0.0117
Full term LGA	0.79 (0.64 – 0.97)	0.024
Preterm SGA	1.97 (1.13 – 3.43)	0.017
Preterm LGA	0.83 (0.45 – 1.56)	0.57
**Elevated ALT**		
Full term SGA	1.26 (1.15 – 1.39)	<.0001
Full term LGA	0.87 (0.80 – 0.95)	0.0011
Preterm SGA	0.96 (0.73 – 1.26)	0.75
Preterm LGA	1.16 (0.92 – 1.48)	0.22

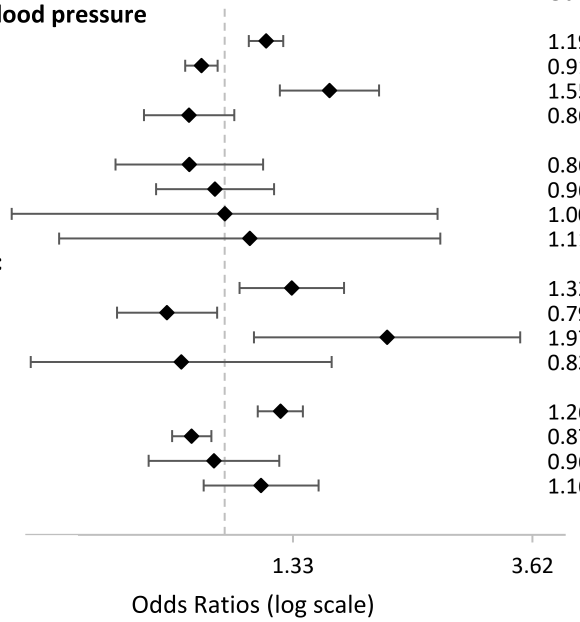

	Odds Ratio (95% CI)	p-value
**Elevated total cholesterol**		
Full term SGA	1.00 (0.89 – 1.12)	0.99
Full term LGA	0.93 (0.84 – 1.03)	0.17
Preterm SGA	1.30 (0.94 – 1.79)	0.11
Preterm LGA	1.02 (0.76 – 1.36)	0.91
**Elevated LDL**		
Full term SGA	1.04 (0.92 – 1.16)	0.55
Full term LGA	1.01 (0.91 – 1.11)	0.93
Preterm SGA	1.39 (1.02 – 1.90)	0.038
Preterm LGA	0.82 (0.60 – 1.11)	0.19
**Low HDL**		
Full term SGA	1.21 (1.10 – 1.33)	<.0001
Full term LGA	0.98 (0.90 – 1.07)	0.68
Preterm SGA	1.03 (0.77 – 1.37)	0.85
Preterm LGA	1.01 (0.78 – 1.29)	0.96
**Elevated Triglycerides**		
Full term SGA	1.11 (1.01 – 1.21)	0.025
Full term LGA	0.95 (0.88 – 1.03)	0.22
Preterm SGA	0.91 (0.70 – 1.18)	0.46
Preterm LGA	0.80 (0.64 – 1.01)	0.057

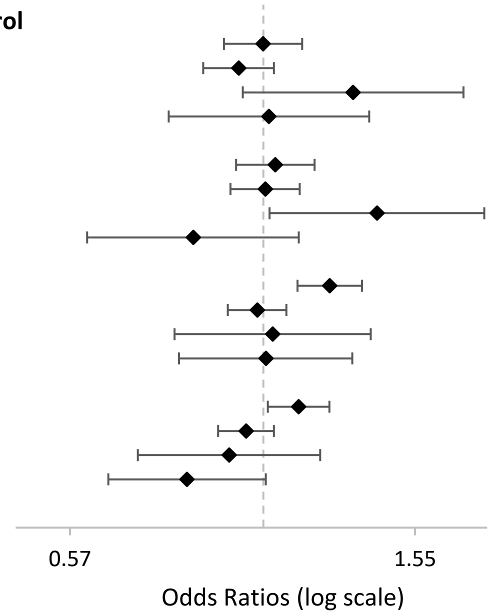

Supplementary Figure 1. Odds ratios (OR) stratified for gestational age, for SGA and LGA with AGA as reference, using Maršál et al [5], from logistic regression adjusted for sex, age group, degree of obesity [11,12], immigration, and obesity register (APV or BORIS). Results are presented with confidence intervals (CI) and p-values. Dashed line indicates OR=1.00.

ALT, alanine aminotransferase; AGA, appropriate for gestational age; BP, blood pressure; HbA1c, glycated hemoglobin; HDL, high density lipoprotein; IFG, impaired fasting glycemia; LDL, low density lipoprotein; LGA, large for gestational age; OR, odds ratio; SDS, standard deviation score; SGA, small for gestational age

Systolic and diastolic blood pressure

Of 38 553 individuals with reported blood pressure, systolic and diastolic hypertensive blood pressure was present in 26.3% and 12.4% respectively. Any hypertensive blood pressure was present in 30.7%. Individuals born SGA were more likely to have both systolic and diastolic blood pressure, while individuals born LGA only had a decreased risk for systolic but not diastolic hypertensive blood pressure, see Supplementary Figure 2 below.

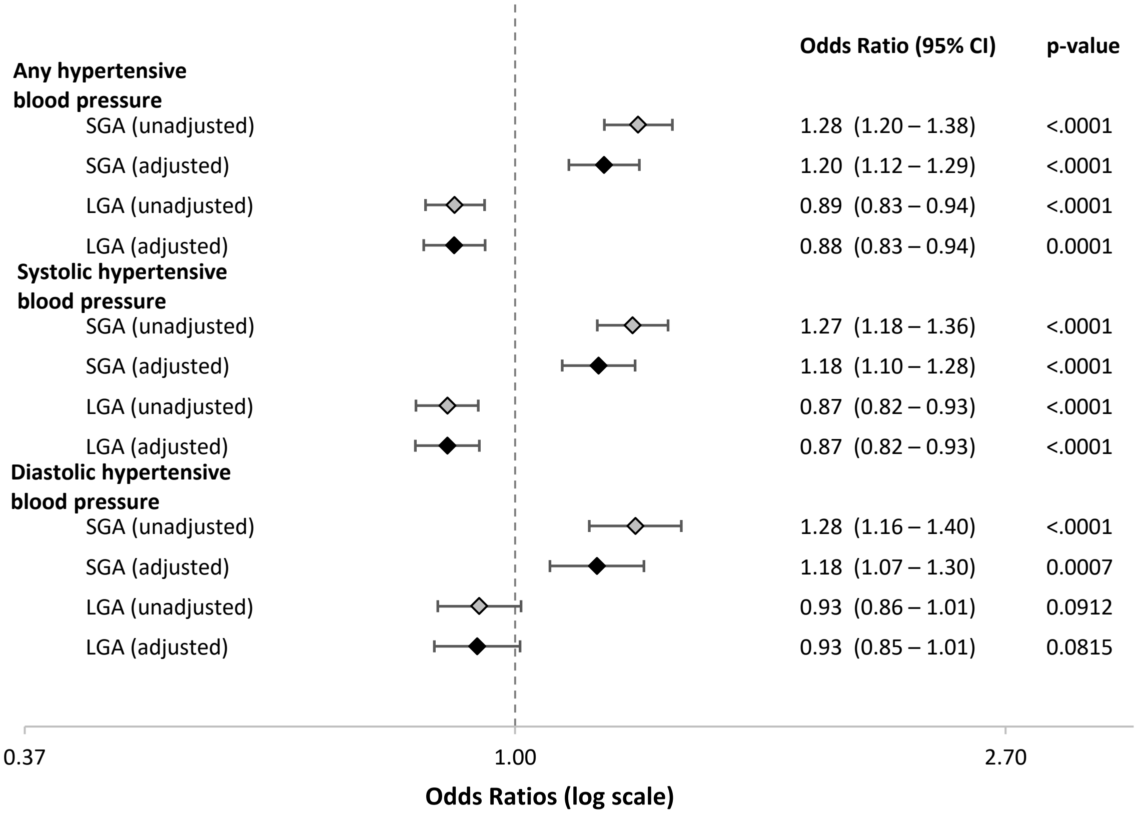

Supplementary Figure 2. Odds ratios (OR) and 95% confidence intervals (CI) for systolic and diastolic blood pressure from logistic regression. Grey markers are unadjusted OR and black markers are OR adjusted for sex, age group, degree of obesity [11,12], immigration, and obesity register (APV or BORIS). Dashed line indicates OR=1.00.

Supplementary Table 5. Odds ratios (OR), 95% confidence intervals (CI), and p-values stratified for sex from logistic regression adjusted for age group, degree of obesity[11,12], immigration, and obesity register (APV or BORIS).

		**Hypertensive blood pressure n = 38 553**		**Impaired fasting glycemia n = 25 200**		**Elevated HbA1c n = 9 247**		**Elevated ALT n = 21 297**		**Elevated total cholesterol n = 28 218**		**Elevated LDL n = 26 344**		**Low HDL n = 26 011**		**Elevated Triglycerides n = 28 004**	
**Strata**		**OR (CI)**	**p**	**OR (CI)**	**p**	**OR (CI)**	**p**	**OR (CI)**	**p**	**OR (CI)**	**p**	**OR (CI)**	**p**	**OR (CI)**	**p**	**OR (CI)**	**p**
	**German reference**[4]																
**Female**	**LGA vs. AGA**	0.87 (0.79 – 0.94)	<0.001	1.14 (0.84 – 1.54)	0.340	0.81 (0.63 – 1.05)	0.108	0.94 (0.85 – 1.04)	0.238	0.98 (0.86 – 1.11)	0.727	0.97 (0.85 – 1.10)	0.850	0.92 (0.83 – 1.02)	0.129	0.93 (0.84 – 1.02)	0.133
**Male**	**LGA vs. AGA**	0.91 (0.83 – 0.99)	0.032	0.73 (0.52 – 1.02)	0.064	0.80 (0.61 – 1.05)	0.102	0.80 (0.71 – 0.89)	<0.001	0.94 (0.83 – 1.08)	0.399	1.00 (0.88 – 1.15)	0.952	1.02 (0.91 – 1.15)	0.714	0.86 (0.77 – 0.96)	0.007
**Female**	**SGA vs. AGA**	1.17 (1.06 – 1.30)	0.002	0.83 (0.52 – 1.33)	0.443	1.58 (1.17 – 2.13)	0.003	1.24 (1.09 – 1.41)	0.001	1.08 (0.92 – 1.27)	0.327	1.02 (0.87 – 1.20)	0.968	1.19 (1.05 – 1.36)	0.008	1.09 (0.97 – 1.24)	0.160
**Male**	**SGA vs. AGA**	1.23 (1.11 – 1.36)	<0.001	1.00 (0.66 – 1.52)	0.997	1.08 (0.77 – 1.53)	0.650	1.18 (1.03 – 1.36)	0.019	1.03 (0.88 – 1.21)	0.720	1.15 (0.98 – 1.34)	0.086	1.22 (1.06 – 1.40)	0.005	1.08 (0.95 – 1.23)	0.236
	**Swedish reference**[5]																
**Female**	**LGA vs. AGA**	0.88 (0.80 – 0.96)	0.001	1.09 (0.79 – 1.51)	0.470	0.75 (0.56 – 0.99)	0.046	0.95 (0.86 – 1.06)	0.486	0.95 (0.83 – 1.09)	0.482	0.98 (0.85 – 1.12)	0.725	0.91 (0.81 – 1.02)	0.092	0.94 (0.85 – 1.05)	0.490
**Male**	**LGA vs. AGA**	0.93 (0.85 – 1.02)	0.128	0.84 (0.60 – 1.18)	0.479	0.83 (0.63 – 1.09)	0.193	0.85 (0.76 – 0.95)	0.006	0.92 (0.80 – 1.05)	0.214	0.97 (0.85 – 1.11)	0.897	1.05 (0.94 – 1.18)	0.645	0.93 (0.83 – 1.04)	0.394
**Female**	**SGA vs. AGA**	1.18 (1.08 – 1.30)	<0.001	0.78 (0.51 – 1.19)	0.236	1.70 (1.31 – 2.20)	<0.001	1.23 (1.09 – 1.38)	<0.001	1.03 (0.89 – 1.19)	0.694	1.03 (0.89 – 1.19)	0.701	1.15 (1.08 – 1.29)	0.025	1.09 (0.97 – 1.22)	0.315
**Male**	**SGA vs. AGA**	1.29 (1.17 – 1.43)	<0.001	0.98 (0.66 – 1.45)	0.927	1.15 (0.84 – 1.58)	0.375	1.24 (1.08 – 1.41)	<0.001	1.03 (0.88 – 1.20)	0.693	1.14 (0.98 – 1.32)	0.226	1.25 (1.10 – 1.43)	0.003	1.11 (0.98 – 1.25)	0.264

Hypertensive blood pressure [6]: Any blood pressure ≥95 percentile for age, sex, and height

Impaired fasting glycemia [7]: ≥6.1 mmol/l respectively 110 mg/dl

Elevated HbA1c [8]: ≥39 mmol/mol respectively 5.7%

Elevated ALT [9]: ≥24 U/l respectively 0.4 µkat/l

Altered lipids [10]: Cholesterol ≥200 mg/dl; LDL ≥130 mg/dl; HDL <40 mg/dl; TG ≥130 mg/dl (10 – 19 years) or ≥100 mg/dl (0 – 9 years)
ALT, alanine aminotransferase; AGA, appropriate for gestational age; HbA1c, glycated hemoglobin; HDL, high density lipoprotein; LDL, low density lipoprotein; LGA, large for gestational age; OR, odds ratio; SGA, small for gestational age

Supplementary Table 6. Odds ratios (OR), 95% confidence intervals (CI), and P values stratified for age group from logistic regression adjusted for sex, degree of obesity [11,12], immigration, and obesity register (APV or BORIS).

		**Hypertensive blood pressure n = 38 553**		**Impaired fasting glycemia n = 25 200**		**Elevated HbA1c n = 9 247**		**Elevated ALT n = 21 297**		**Elevated total cholesterol n = 28 218**		**Elevated LDL n = 26 344**		**Low HDL n = 26 011**		**Elevated Triglycerides n = 28 004**	
		**OR (CI)**	**P**	**OR (CI)**	**p**	**OR (CI)**	**p**	**OR (CI)**	**p**	**OR (CI)**	**p**	**OR (CI)**	**p**	**OR (CI)**	**p**	**OR (CI)**	**p**
	**German reference**[4]																
**<11y**	**LGA vs. AGA**	0.85 (0.77 – 0.95)	0.006	0.85 (0.56 – 1.30)	0.456	0.77 (0.58 – 1.03)	0.082	0.91 (0.81 – 1.01)	0.084	1.07 (0.93 – 1.22)	0.352	1.01 (0.88 – 1.16)	0.844	0.90 (0.78 – 1.03)	0.124	0.94 (0.84 – 1.06)	0.326
**11-14y**	**LGA vs. AGA**	0.93 (0.84 – 1.03)	0.173	0.92 (0.63 – 1.34)	0.660	0.71 (0.51 – 0.99)	0.041	0.82 (0.72 – 0.93)	0.003	0.89 (0.75 – 1.05)	0.163	1.00 (0.85 – 1.18)	0.965	1.00 (0.87 – 1.14)	0.956	0.85 (0.74 – 0.97)	0.017
**≥14y**	**LGA vs. AGA**	0.87 (0.77 – 0.98)	0.019	1.01 (0.69 – 1.47)	0.915	1.05 (0.73 – 1.51)	0.814	0.87 (0.74 – 1.02)	0.087	0.86 (0.71 – 1.05)	0.145	0.90 (0.74 – 1.08)	0.261	1.02 (0.89 – 1.18)	0.749	0.88 (0.76 – 1.02)	0.033
**<11y**	**SGA v. AGA**	1.25 (1.11 – 1.41)	<0.001	1.24 (0.72 – 2.15)	0.440	1.56 (1.10 – 2.21)	0.012	1.36 (1.16 – 1.60)	<0.001	1.12 (0.94 – 1.34)	0.219	1.11 (0.92 – 1.34)	0.280	1.17 (0.98 – 1.40)	0.090	1.13 (0.98 – 1.32)	0.101
**11-14y**	**SGA v. AGA**	1.20 (1.07 – 1.35)	0.002	0.80 (0.47 – 1.37)	0.418	0.95 (0.63 – 1.44)	0.805	1.13 (0.96 – 1.33)	0.133	1.00 (0.82 – 1.21)	0.985	1.14 (0.94 – 1.38)	0.182	1.21 (1.04 – 1.40)	0.016	1.04 (0.89 – 1.21)	0.642
**≥14y**	**SGA v. AGA**	1.15 (1.01 – 1.31)	0.039	0.84 (0.50 – 1.42)	0.515	1.54 (1.02 – 2.34)	0.041	1.14 (0.95 – 1.37)	0.148	1.05 (0.85 – 1.30)	0.666	1.00 (0.81 – 1.24)	0.972	1.24 (1.05 – 1.46)	0.010	1.10 (0.93 – 1.30)	0.286
	**Swedish reference**[5]																
**<11y**	**LGA vs. AGA**	0.89 (0.80 – 0.99)	0.084	0.84 (0.53 – 1.30)	0.427	0.84 (0.62 – 1.14)	0.253	0.94 (0.84 – 1.06)	0.309	1.03(0.90 – 1.19)	0.656	1.00 (0.86 – 1.15)	0.976	0.93 (0.80 – 1.07)	0.280	0.99 (0.88 – 1.11)	0.835
**11-14y**	**LGA vs. AGA**	0.93 (0.83 – 1.03)	0.157	0.97 (0.65 – 1.44)	0.869	0.62 (0.43 – 0.89)	0.025	0.83 (0.73 – 0.95)	0.008	0.85 (0.72 – 1.01)	0.068	0.97 (0.82 – 1.15)	0.707	1.02 (0.89 – 1.17)	0.948	0.88 (0.77 – 1.01)	0.059
**≥14y**	**LGA vs. AGA**	0.88 (0.78 – 0.995)	0.041	1.08 (0.73 – 1.58)	0.712	1.00 (0.68 – 1.47)	0.989	0.92 (0.78 – 1.08)	0.306	0.86 (0.71 – 1.05)	0.138	0.93 (0.77 – 1.12)	0.441	0.99 (0.85 – 1.14)	0.980	0.94 (0.81 – 1.09)	0.376
**<11y**	**SGA v. AGA**	1.32 (1.18 – 1.48)	<0.001	1.03 (0.61 – 1.76)	0.904	1.79 (1.32 – 2.43)	<0.001	1.38 (1.20 – 1.59)	<0.001	1.10 (0.93 – 1.30)	0.264	1.09 (0.92 – 1.30)	0.325	1.19 (1.01 – 1.40)	0.038	1.09 (0.95 – 1.25)	0.232
**11-14y**	**SGA v. AGA**	1.23 (1.10 – 1.38)	<0.001	0.90 (0.56 – 1.44)	0.649	1.08 (0.75 – 1.55)	0.914	1.16 (1.00 – 1.35)	0.047	0.96 (0.80 – 1.16)	0.686	1.08 (0.90 – 1.30)	0.658	1.18 (1.02 – 1.36)	0.026	1.10 (0.95 – 1.27)	0.187
**≥14y**	**SGA v. AGA**	1.14 (1.00 – 1.29)	0.046	0.74 (0.45 – 1.23)	0.244	1.49 (1.02 – 2.18)	0.041	1.11 (0.94 – 1.32)	0.223	1.02 (0.83 – 1.25)	0.863	1.07 (0.87 – 1.30)	0.533	1.20 (1.03 – 1.40)	0.022	1.09(0.93 – 1.28)	0.298

Hypertensive blood pressure [6]: Any blood pressure ≥95 percentile for age, sex, and height

Impaired fasting glycemia [7]: ≥6.1 mmol/l respectively 110 mg/dl

Elevated HbA1c [8]: ≥39 mmol/mol respectively 5.7%

Elevated ALT [9]: ≥24 U/l respectively 0.4 µkat/l

Altered lipids [10]: Cholesterol ≥200 mg/dl; LDL ≥130 mg/dl; HDL <40 mg/dl; TG ≥130 mg/dl (10 – 19 years) or ≥100 mg/dl (0 – 9 years)
ALT, alanine aminotransferase; AGA, appropriate for gestational age; CL, 95% confidence limit; HbA1c, glycated hemoglobin; HDL, high density lipoprotein; LDL, low density lipoprotein; LGA, large for gestational age; OR, odds ratio; SGA, small for gestational age

Supplementary Table 7. Odds ratios (OR), 95% confidence intervals (CI), and p-values stratified for weight status from logistic regression adjusted for sex, age group, immigration, and obesity register (APV or BORIS).

		**Hypertension n = 38 553**		**Impaired fasting glycemia n = 25 200**		**Elevated HbA1c n = 9 247**		**Elevated ALT n = 21 297**		**Elevated total cholesterol n = 28 218**		**Elevated LDL n = 26 344**		**Low HDL n = 26 011**		**Elevated Triglycerides n = 28 004**	
**Strata**		**OR (CI)**	**P**	**OR (CI)**	**p**	**OR (CI)**	**p**	**OR (CI)**	**p**	**OR (CI)**	**p**	**OR (CI)**	**p**	**OR (CI)**	**p**	**OR (CI)**	**p**
	**German reference**[4]																
**Ow**	**LGA vs. AGA**	0.83 (0.67 – 1.04)	0.098	1.06 (0.53 – 2.12)	0.868	0.28 (0.10 – 0.78)	0.015	0.71 (0.56 – 0.90)	0.005	0.67 (0.49 – 0.91)	0.011	0.79 (0.58 – 1.07)	0.129	1.25 (0.96 – 1.62)	0.101	0.77 (0.59 – 0.99)	0.042
**Ob I**	**LGA vs. AGA**	0.90 (0.81 – 0.99)	0.030	0.76 (0.53 – 1.11)	0.157	0.91 (0.69 – 1.22)	0.536	0.85 (0.76 – 0.95)	0.01	0.96 (0.84 – 1.10)	0.542	0.96 (0.83 – 1.10)	0.523	0.91 (0.80 – 1.03)	0.122	0.89 (0.79 – 1.00)	0.050
**Ob II**	**LGA vs. AGA**	0.86 (0.77 – 0.97)	0.011	1.10 (0.72 – 1.69)	0.655	0.84 (0.58 – 1.14)	0.233	0.98 (0.85 – 1.13)	0.797	1.06 (0.89 – 1.27)	0.506	1.10 (0.93 – 1.31)	0.284	1.00 (0.87 – 1.15)	0.983	0.88 (0.77 – 1.01)	0.072
**Ob III**	**LGA vs. AGA**	0.92 (0.80 – 1.05)	0.207	0.95 (0.61 – 1.48)	0.823	0.82 (0.56 – 1.22)	0.325	0.90 (0.74 – 1.08)	0.242	1.03 (0.82 – 1.30)	0.794	1.00 (0.80 – 1.25)	0.994	0.95 (0.80 – 1.14)	0.595	1.01 (0.85 – 1.19)	0.945
**Ow**	**SGA v. AGA**	1.18 (0.98 – 1.43)	0.082	1.37 (0.69 – 2.75)	0.368	1.00 (0.50 – 2.02)	0.996	1.55 (1.22 – 1.97)	<0.001	1.07 (0.82 – 1.40)	0.610	1.13 (0.86 – 1.50)	0.384	1.10 (0.83 – 1.45)	0.510	1.07 (0.85 – 1.35)	0.563
**Ob I**	**SGA v. AGA**	1.21 (1.09 – 1.35)	<0.001	1.12 (0.73 – 1.74)	0.600	1.56 (1.12 – 2.18)	0.009	1.18 (1.03 – 1.35)	0.051	1.09 (0.93 – 1.28)	0.304	1.05 (0.89 – 1.24)	0.567	1.19 (1.03 – 1.37)	0.018	1.05 (0.91 – 1.20)	0.510
**Ob II**	**SGA v. AGA**	1.20 (1.05 – 1.38)	0.009	0.59 (0.27 – 1.29)	0.185	1.43 (0.95 – 2.15)	0.089	1.04 (0.86 – 1.25)	0.701	0.99 (0.78 – 1.25)	0.903	1.06 (0.84 – 1.33)	0.648	1.24 (1.04 – 1.48)	0.017	1.07 (0.90 – 1.27)	0.459
**Ob III**	**SGA v. AGA**	1.17 (0.97 – 1.41)	0.099	0.52 (0.21 – 1.29)	0.157	0.96 (0.53 – 1.74)	0.888	1.44 (1.06 – 1.95)	0.020	1.06 (0.76 – 1.47)	0.738	1.22 (0.90 – 1.66)	0.200	1.22 (0.96 – 1.55)	0.097	1.26 (1.00 – 1.58)	0.050
	**Swedish reference**[5]																
**Ow**	**LGA vs. AGA**	0.84 (0.68 – 1.05)	0.113	1.39 (0.71 – 2.70)	0.337	0.58 (0.26 – 1.29)	0.181	0.79 (0.62 – 1.01)	0.055	0.63 (0.46 – 0.87)	0.004	0.79 (0.58 – 1.08)	0.132	1.26 (0.97 – 1.64)	0.087	0.78 (0.60 – 1.01)	0.056
**Ob I**	**LGA vs. AGA**	0.92 (0.83 – 1.01)	0.086	0.82 (0.55 – 1.21)	0.313	0.72 (0.52 – 0.99)	0.042	0.87 (0.78 – 0.98)	0.017	0.94 (0.81 – 1.08)	0.361	0.94 (0.82 – 1.09)	0.421	0.91 (0.80 – 1.04)	0.154	0.97 (0.86 – 1.09)	0.602
**Ob II**	**LGA vs. AGA**	0.88 (0.79 – 0.99)	0.034	0.98 (0.62 – 1.54)	0.928	0.87 (0.61 – 1.25)	0.454	0.98 (0.84 – 1.13)	0.749	1.00 (0.83 – 1.20)	0.999	1.06 (0.89 – 1.27)	0.510	1.03 (0.89 – 1.19)	0.721	0.91 (0.79 – 1.05)	0.184
**Ob III**	**LGA vs. AGA**	0.93 (0.81 – 1.07)	0.303	0.97 (0.61 – 1.54)	0.992	0.92 (0.61 – 1.38)	0.674	0.98 (0.81 – 1.19)	0.829	1.05 (0.83 – 1.32)	0.710	1.04 (0.83 – 1.30)	0.753	0.95 (0.79 – 1.13)	0.532	1.00 (0.84 – 1.19)	0.981
**Ow**	**SGA v. AGA**	1.23 (1.03 – 1.47)	0.035	1.53 (0.80 – 2.92)	0.196	1.31 (0.72 – 2.39)	0.369	1.67 (1.33 – 2.10)	<0.001	1.05 (0.82 – 1.36)	0.698	1.14 (0.87 – 1.49)	0.352	1.13 (0.87 – 1.47)	0.366	1.14 (0.91 – 1.42)	0.256
**Ob I**	**SGA v. AGA**	1.24 (1.12 – 1.37)	<0.001	1.02 (0.67 – 1.54)	0.933	1.49 (1.09 – 2.02)	0.011	1.18 (1.04 – 1.34)	0.011	1.06 (0.91 – 1.24)	0.449	1.01 (0.86 – 1.18)	0.920	1.17 (1.02 – 1.34)	0.021	1.03 (0.91 – 1.17)	0.672
**Ob II**	**SGA v. AGA**	1.23 (1.08 – 1.40)	0.035	0.47 (0.22 – 1.01)	0.054	1.65 (1.15 – 2.36)	0.007	1.04 (0.87 – 1.23)	0.699	0.94 (0.75 – 1.18)	0.582	1.10 (0.89 – 1.37)	0.378	1.19 (1.01 – 1.41)	0.040	1.09 (0.92 – 1.29)	0.306
**Ob III**	**SGA v. AGA**	1.22 (1.02 – 1.45)	0.026	0.62 (0.30 – 1.31)	0.423	1.15 (0.69 – 1.92)	0.601	1.41 (1.07 – 1.85)	0.015	1.07 (0.79 – 1.45)	0.682	1.25 (0.94 – 1.66)	0.118	1.27 (1.02 – 1.59)	0.035	1.27 (1.02 – 1.57)	0.030

Hypertensive blood pressure [6]: Any blood pressure ≥95 percentile for age, sex, and height
Impaired fasting glycemia [7]: ≥6.1 mmol/l respectively 110 mg/dl
Elevated HbA1c [8]: ≥39 mmol/mol respectively 5.7%
Elevated ALT [9]: ≥24 U/l respectively 0.4 µkat/l
Altered lipids [10]: Cholesterol ≥200 mg/dl; LDL ≥130 mg/dl; HDL <40 mg/dl; TG ≥130 mg/dl (10 – 19 years) or ≥100 mg/dl (0 – 9 years)
ALT, alanine aminotransferase; AGA, appropriate for gestational age; CL, 95% confidence limit; HbA1c, glycated hemoglobin; HDL, high density lipoprotein; LDL, low density lipoprotein; LGA, large for gestational age; Ob I, obesity class I; Ob II, obesity class II; Ob III, obesity class III; OR, odds ratio; Ow, overweight; SGA, small for gestational age

Supplementary Table 8. Odds ratios (OR), 95% confidence intervals (CI), and P values stratified for stratified for obesity register from logistic regression adjusted for sex, age group, degree of obesity [11,12], and immigration

Strata		Hypertensive blood pressure		Impaired fasting glycemia		Elevated HbA1c		Elevated ALT		Elevated total cholesterol		Elevated LDL		Low HDL		Elevated Triglycerides	
		**OR (CI)**	**p**	**OR (CI)**	**p**	**OR (CI)**	**p**	**OR (CI)**	**p**	**OR (CI)**	**p**	**OR (CI)**	**p**	**OR (CI)**	**p**	**OR (CI)**	**p**
**APV**	**LGA vs. AGA**	0.91 (0.84 – 0.97)	0.007	0.92 (0.65 – 1.32)	0.660	0.83 (0.62 – 1.11)	0.217	0.85 (0.77 – 0.94)	0.002	0.97 (0.86 – 1.09)	0.844	0.98 (0.87 – 1.10)	0.758	0.93 (0.84 – 1.03)	0.161	0.90 (0.83 – 0.99)	0.031
**BORIS**	**LGA vs. AGA**	0.82 (0.72 – 0.93)	0.003	0.94 (0.71 – 1.26)	0.699	0.80 (0.63 – 1.02)	0.069	0.90 (0.80 – 1.00)	0.050	0.95 (0.81 – 1.11)	0.500	0.98 (0.84 – 1.13)	0.763	1.04 (0.91 – 1.18)	0.557	0.89 (0.78 – 1.02)	0.095
**APV**	**SGA vs. AGA**	1.16 (1.08 – 1.25)	<0.001	0.66 (0.42 – 1.04)	0.072	1.47 (1.11 – 1.95)	0.007	1.19 (1.07 – 1.33)	0.002	1.06 (0.93 – 1.19)	0.667	1.11 (0.98 – 1.26)	0.099	1.17 (1.05 – 1.29)	0.004	1.04 (0.94 – 1.15)	0.400
**BORIS**	**SGA vs. AGA**	1.50 (1.23 – 1.82)	<0.001	1.35 (0.88 – 2.07)	0.177	1.11 (0.76 – 1.62)	0.600	1.26 (1.04 – 1.52)	0.020	1.06 (0.81 – 1.11)	0.668	0.98 (0.75 – 1.28)	0.869	1.31 (1.05 – 1.63)	0.017	1.30 (1.05 – 1.61)	0.018

Hypertensive blood pressure [6]: Any blood pressure ≥95 percentile for age, sex, and height

Impaired fasting glycemia [7]: ≥6.1 mmol/l respectively 110 mg/dl

Elevated HbA1c [8]: ≥39 mmol/mol respectively 5.7%

Elevated ALT [9]: ≥24 U/l respectively 0.4 µkat/l

Altered lipids [10]: Cholesterol ≥200 mg/dl; LDL ≥130 mg/dl; HDL <40 mg/dl; TG ≥130 mg/dl (10 – 19 years) or ≥100 mg/dl (0 – 9 years)

ALT, alanine aminotransferase; AGA, appropriate for gestational age; CL, 95% confidence limit; HbA1c, glycated hemoglobin; HDL, high density lipoprotein; LDL, low density lipoprotein; LGA, large for gestational age; OR, odds ratio; SGA, small for gestational age

References

1. Martin L, Oepen J, Reinehr T, Wabitsch M, Claussnitzer G, Waldeck E, et al. Ethnicity and cardiovascular risk factors: evaluation of 40,921 normal-weight, overweight or obese children and adolescents living in Central Europe. Int J Obes (Lond). 2015;39(1):45-51. Epub 2014/09/13. doi: 10.1038/ijo.2014.167. PubMed PMID: 25214149.
2. Bohn B, Stachow R, Gellhaus I, Matthias J, Lichtenstern H, Holl RW. Heterogeneity in sociodemographic characteristics and cardiovascular risk factors at the initiation of a lifestyle intervention for obesity within Germany: an APV multicenter study on 40,942 children and adolescents. Child and Adolescent Obesity. 2018;1(1):5-17. doi: 10.1080/2574254x.2018.1547070.
3. Hagman E, Danielsson P, Lindberg L, Marcus C, Committee BS. Paediatric obesity treatment during 14 years in Sweden: Lessons from the Swedish Childhood Obesity Treatment Register-BORIS. Pediatr Obes. 2020;15(7):e12626. Epub 2020/02/20. doi: 10.1111/ijpo.12626. PubMed PMID: 32074662.
4. Voigt M, Rochow N, Schneider KT, Hagenah HP, Scholz R, Hesse V, et al. [New percentile values for the anthropometric dimensions of singleton neonates: analysis of perinatal survey data of 2007-2011 from all 16 states of Germany]. Z Geburtshilfe Neonatol. 2014;218(5):210-7. Epub 2014/10/30. doi: 10.1055/s-0034-1385857. PubMed PMID: 25353215.
5. Marsal K, Persson PH, Larsen T, Lilja H, Selbing A, Sultan B. Intrauterine growth curves based on ultrasonically estimated foetal weights. Acta Paediatr. 1996;85(7):843-8. Epub 1996/07/01. doi: 10.1111/j.1651-2227.1996.tb14164.x. PubMed PMID: 8819552.
6. National High Blood Pressure Education Program Working Group on High Blood Pressure in C, Adolescents. The fourth report on the diagnosis, evaluation, and treatment of high blood pressure in children and adolescents. Pediatrics. 2004;114(2 Suppl 4th Report):555-76. Epub 2004/08/03. PubMed PMID: 15286277.
7. World Health Oganization G, Switzerland. Definition and diagnosis of diabetes mellitus and intermediate hyperglycemia: report of a WHO/IDF Consultation. 2006.
8. American Diabetes A. Diagnosis and classification of diabetes mellitus. Diabetes Care. 2011;34 Suppl 1:S62-9. Epub 2011/01/14. doi: 10.2337/dc11-S062. PubMed PMID: 21193628; PubMed Central PMCID: PMCPMC3006051.
9. Koutny F, Weghuber D, Bollow E, Greber-Platzer S, Hartmann K, Korner A, et al. Prevalence of prediabetes and type 2 diabetes in children with obesity and increased transaminases in European German-speaking countries. Analysis of the APV initiative. Pediatr Obes. 2020;15(4):e12601. Epub 2019/12/07. doi: 10.1111/ijpo.12601. PubMed PMID: 31810110; PubMed Central PMCID: PMCPMC7079233.
10. Expert Panel on Integrated Guidelines for Cardiovascular H, Risk Reduction in C, Adolescents, National Heart L, Blood I. Expert panel on integrated guidelines for cardiovascular health and risk reduction in children and adolescents: summary report. Pediatrics. 2011;128 Suppl 5:S213-56. Epub 2011/11/16. doi: 10.1542/peds.2009-2107C. PubMed PMID: 22084329; PubMed Central PMCID: PMCPMC4536582.
11. Cole TJ, Lobstein T. Extended international (IOTF) body mass index cut-offs for thinness, overweight and obesity. Pediatr Obes. 2012;7(4):284-94. Epub 2012/06/21. doi: 10.1111/j.2047-6310.2012.00064.x. PubMed PMID: 22715120.
12. Bervoets L, Massa G. Defining morbid obesity in children based on BMI 40 at age 18 using the extended international (IOTF) cut-offs. Pediatr Obes. 2014;9(5):e94-8. Epub 2014/03/01. doi: 10.1111/j.2047-6310.2014.00217.x. PubMed PMID: 24578314.
