# Supplementary material for: The association between perinatal factors and cardiometabolic risk factors in children and adolescents with overweight or obesity: A retrospective two-cohort study
Source: PLoS Med. 2023 Jan 13;20(1):e1004165. doi: 10.1371/journal.pmed.1004165 (PMC9886302; doi:10.1371/journal.pmed.1004165)
Supplement: S3 File — (DOCX) [file pmed.1004165.s003.docx]

# APV – BORIS collaboration

*Perinatal factors and later risk for cardio-metabolic derangements in children and adolescents with obesity*

**Background:** Being born small for gestational age (SGA) increases the risk for cardio-metabolic disease in adulthood. While being born large for gestational age (LGA) increases the risk for obesity in childhood and SGA-born children have lower risk for obesity in childhood, it remains unknown if children with obesity and born SGA have higher risk for cardio-metabolic derangements.

**Question:** Do perinatal factors affect the risk for cardio-metabolic derangements in children and adolescents with obesity?

**Setting:** Longitudinal data from the pediatric obesity registers BORIS linked with national registers from Sweden and APV with data from Germany, Austria and Switzerland.

| Approximate no. of individuals with birth data and cardio-metabolic markers | | | |
| --- | --- | --- | --- |
|  | BORIS | APV | Total |
| Blood pressure | 8 000 | 37 000 | 45 000 |
| Fasting glucose | 9 000 | 26 000 | 35 000 |
| HbA1c | 7 000 | 9 000 | 16 000 |
| Liver enzymes | 9 000 | 19 000 | 28 000 |
| Lipids | 9 000 | 27 000 | 36 000 |

**Exposure:** Two perinatal factors will be evaluated: Weight for gestational age and gestational age (reference for these two are TBD).

**Outcome:** Deranged blood pressure, fasting glucose, HbA1c, liver enzymes (ALT and/or AST), lipids (triglycerides, total cholesterol, HDL and LDL) at the first documented visit.

**Covariates:** Sex (ref female), degree of obesity defined by IOTF (ref obesity class I), age category (ref lowest age group), migration background (ref non-immigrant), register source (ref APV).

**Inclusion criteria:** Patients with overweight or obesity according to IOTF, up to 18 years of age at obesity treatment initiation and with data of the outcome of interest (see variables above) are eligible for inclusion.

**Exclusion criteria:**  Obesity associated syndromes (e.g. Prader Willi and LMBB), and other genetic syndromes (e.g. Mb. Down, Turner, Fragile X, Klinefelter) will be excluded. If possible, medical drugs or conditions affecting the exposure or the outcome.

**Research data and data sets:** Data set to be sent from Sweden will be named “BORIS_data_to_Ulm”. The research and transfer of data is approved according to the Swedish Ethical Review Authority, file no: 2016/922-31/1, amendment 2020-05646.

**Statistical analysis plan:**

- Descriptive description of the cohorts; Stratification of patients according to weight for gestational age.

- To evaluate the risk for co-morbidities, multivariable regression analyses (logistic models) with age categories, sex, migration background, degree of obesity, and register source as covariates will be applied. Results will be presented with 95% confidence limits.

- Stratification for age categories, sex, degree of obesity, and register source will give extra information and will be performed if the study material has power to allow such analyses.
